# Zero-shot benchmarking of RNA language models in structural, functional, and evolutionary learning

**DOI:** 10.1093/bib/bbag098

**Published:** 2026-03-06

**Authors:** He Wang, Yikun Zhang, Jie Chen, Jian Zhan, Yaoqi Zhou

**Affiliations:** Institute of Systems and Physical Biology, Shenzhen Bay Laboratory, Weiguang Life Sciences Park, Xinhu Street, Guangming District, Shenzhen 518107, China; Institute of Systems and Physical Biology, Shenzhen Bay Laboratory, Weiguang Life Sciences Park, Xinhu Street, Guangming District, Shenzhen 518107, China; School of Electronic and Computer Engineering, Peking University, No. 2199 Lishui Road, Nanshan District, Shenzhen 518055, China; School of Electronic and Computer Engineering, Peking University, No. 2199 Lishui Road, Nanshan District, Shenzhen 518055, China; Institute of Systems and Physical Biology, Shenzhen Bay Laboratory, Weiguang Life Sciences Park, Xinhu Street, Guangming District, Shenzhen 518107, China; Ribopeutic (Shenzhen) Co., Ltd., Room 3106-B022, 3038 Jintian Road, Futian District, Shenzhen, Guangdong 518033, China; Ribopeutic Inc., Rm. 517, Bldg. 21, Hexiang Tech Ctr., Qiantang Dist. Hangzhou 310018, Zhejiang Province, China; Institute of Systems and Physical Biology, Shenzhen Bay Laboratory, Weiguang Life Sciences Park, Xinhu Street, Guangming District, Shenzhen 518107, China

**Keywords:** RNA language models, zero-shot evaluation, RNA secondary structure, RNA classification, mutational fitness prediction

## Abstract

RNA language models (LMs) are increasingly applied to RNA structure and function analysis, yet their intrinsic representational capacities remain poorly characterized. Here, we present a standardized zero-shot evaluation of 21 RNA LMs, with representative DNA LMs included as reference controls. Three complementary tasks—attention-based RNA secondary structure prediction, embedding-based RNA classification, and mutational fitness estimation from sequence likelihoods—are evaluated without downstream fine-tuning. Our results reveal substantial variability across models and clear trade-offs between structural, functional, and evolutionary representations. RNA-specific, noncoding RNA-enriched pretraining is crucial for capturing structural information, while evolutionary signals from multiple sequence alignments substantially boost performance. Although model scaling yields gains, architectural and objective choices critically influence performance across task categories. Together, this study provides a foundational benchmark, highlights inherent challenges in learning unified RNA representations, and offers insights for developing next-generation RNA foundation models.

## Introduction

RNA molecules play central roles in virtually all aspects of cellular regulation, ranging from gene expression control and catalysis to molecular recognition and therapeutic intervention [1–13]. Besides messenger RNAs (mRNAs), diverse classes of noncoding RNAs (ncRNAs)—including ribosomal RNAs, transfer RNAs, microRNAs, long ncRNAs, ribozymes, and regulatory untranslated regions—are now recognized as key functional entities whose activities are intimately linked to their sequence-encoded structural and evolutionary constraints [14–18]. Despite the rapid accumulation of RNA sequence data, experimentally determined RNA structures and functional annotations remain scarce [19, 20], resulting in an expanding gap between sequence availability and biological understanding.

In parallel, large-scale language models (LMs) have transformed natural language processing by learning contextual representations from massive unlabeled corpora. Transformer-based architectures [21], such as BERT [22], GPT [23, 24], and T5 [25], capture long-range dependencies through self-attention and have demonstrated remarkable transferability across downstream tasks. The conceptual analogy between natural language and biological sequences—both composed of discrete tokens governed by context-dependent rules—has motivated the extension of language modeling approaches to proteins, DNA, and RNA [26–43].

Protein language models (PLMs) have experienced particularly rapid development and provide an important precedent for the RNA field. Early foundational models, such as ESM-1b [35] and ProtT5 [37], demonstrated that structural and functional information can emerge from unsupervised training on large protein sequence databases. Subsequent scaling and architectural refinements led to more multimodal PLMs, such as ProtCLIP [44], ProteinGPT [45], and ESM-3 [46], which integrate sequence information with functional or structural signals, enabling improved zero-shot inference and broadening the scope of protein representation learning. Meanwhile, systematic evaluations such as ProteinGym [47] and PEER [48] have highlighted that model architecture, data composition, and evaluation protocols critically influence apparent performance, underscoring the necessity of standardized and task-agnostic benchmarking across different PLMs [49].

Inspired by the success of PLMs, a growing number of RNA LMs have emerged in recent years. Early models, including RNABERT [50] and RNA-FM [26, 51], adopted BERT-style masked language modeling and were pretrained primarily on ncRNA databases. More recent efforts expanded along multiple directions: (i) scaling model size and training data (e.g. Uni-RNA [27], AIDO.RNA [42], and RiNALMo [30]), including the use of the master database of all possible RNA sequences (MARS) [52]; (ii) incorporating structural or motif-level inductive biases during pretraining (e.g. RNAErnie [31], ERNIE-RNA [28], MP-RNA [41], and PlantRNA-FM [53]); (iii) introducing alternative masking strategies (e.g. RNAErnie [31], RNA-km [32]); (iv) adopting alternative positional encoding schemes (e.g. RNA-km [32]); (v) leveraging evolutionary information through multiple sequence alignments (e.g. RNA-MSM [29]); (vi) adopting k-mer or byte pair encoding (BPE) tokenization instead of single-nucleotide representations (e.g. 3UTRBERT [54], BiRNA-BERT [43]); (vii) employing novel pretraining strategies (e.g. ProtRNA [40]); (viii) exploring alternative network architectures (e.g. Mamba2-based DGRNA [55], GPT-based GenerRNA [56], Llama-based RFamLlama [57]); and (ix) developing unified or cross-modal models capable of processing RNA together with DNA and proteins (e.g. LucaOne [58]). These models have been applied to a wide range of downstream tasks, such as RNA secondary structure prediction [26–28, 30–32, 40–43], RNA family and type classification [26, 59], splicing and modification sites analysis [27, 42, 60], RNA–protein/RNA interaction prediction [61, 62], and mutational fitness estimation [57].

Despite the rapid progress of RNA LMs, several fundamental limitations remain in the current landscape of RNA LMs. First, unlike the protein domain—where standardized benchmarks and systematic comparative studies have clarified the strengths and limitations of different model architectures—RNA LMs were typically evaluated on heterogeneous downstream tasks using disparate datasets, fine-tuning strategies, and performance metrics. This fragmentation hampers cross-study comparison and obscures which aspects of RNA biology are intrinsically captured by different models prior to task-specific supervision. Second, although DNA LMs are sometimes adopted as baselines in RNA-related studies, there has been no comprehensive assessment of whether RNA-specific pretraining provides measurable advantages over generic nucleotide LMs [63, 64]. Moreover, in contrast to the large impact of PLMs on protein structure prediction, most notably exemplified by the emergence of AlphaFold [65] and subsequent development of single-sequence-based methods (e.g. ESMfold [66], OmegaFold [67]), it remains unclear whether RNA LMs can lead to a comparable advance in RNA structural modeling; notably, the recent CASP16 assessment failed to reveal significant advancement [68]. Third, RNA LMs differ substantially in network architecture, sequence encoding schemes, and pretraining strategies, yet the influence of these design choices on performance across structurally versus functionally oriented tasks remains poorly understood. Finally, the diversity of RNA training corpora—ranging from ncRNA-focused databases to mRNA-centric transcriptomes and unified multiomics datasets—raises the question of how pretraining scope affects the balance between structural and functional generalization.

A limited number of recent studies have begun to benchmark and evaluate RNA and DNA LMs on specific classes of tasks. These efforts include comprehensive evaluations of six LMs for RNA secondary structure prediction [69], large-scale benchmarks for RNA fitness and structure prediction (e.g. RNAGym, evaluating three RNA LMs [63]), nucleotide foundation model benchmarks focused on fitness prediction (e.g. NABENCH, evaluating ten RNA LMs [70]), and broader task collections for RNA modeling such as BEACON [71] (evaluating seven RNA LMs) and RNA-Scope [64] (evaluating six RNA LMs). While these efforts have provided valuable task-specific insights and established important datasets and evaluation protocols, they typically focus on a single task category or application domain, evaluate a limited subset of RNA LMs, or rely on task-specific fine-tuning or supervised training. Consequently, a comprehensive and standardized analysis that systematically compares a broad spectrum of RNA LMs under identical zero-shot evaluation protocols—while explicitly disentangling structural and functional signals and extensively examining information derived directly from unmodified model outputs—remains lacking.

Here, we present a comprehensive and standardized evaluation of RNA LMs. Using representative DNA LMs as reference controls, we assess 21 RNA LMs across three complementary zero-shot tasks: attention-based RNA secondary structure prediction, embedding-based RNA classification, and RNA fitness prediction from mutational likelihoods. By focusing exclusively on zero-shot settings, we aim to disentangle the intrinsic representational capacity of each model from the effects of downstream fine-tuning. This study provides a unified benchmark for RNA LMs, clarifies trade-offs between structural and functional learning, and highlights key challenges and opportunities for the next generation of RNA foundation models.

## RNA language models and downstream task landscape

### Collection and categorization of RNA language models

To enable a systematic and representative comparison, we first curated a comprehensive collection of RNA LMs from recent literature and publicly available resources over the past 3 years (see Methods for details). In total, 21 RNA LMs were identified within the search period (Table 1). Based on their pretraining data, these models were categorized into three classes. Class I models were trained primarily on diverse RNA datasets, particularly containing large amounts of ncRNAs. Class II models trained exclusively on mRNA-related sequences. Class III models comprised unified or multimodal architectures jointly trained on RNA, DNA, and/or protein sequences. Given the rapid evolution of the field, some recently proposed models or preprints lacking stable public checkpoints may not be included, despite extensive efforts to ensure comprehensive coverage.

**Table 1:** Overview of representative RNA LMs, grouped by pretraining data characteristics, with published models highlighted in bold.

**Class**	**Model**	**Year**	**Reference**	**Parameters**	**Pretraining dataset**	**Input modality**	**Tokenization**	**Architecture**	**Layers**	**Heads**	**Embedding dim.**	**Max input length**
I	**RNA-FM**	2022	[26, 51]	100M	RNAcentral [19] (~23M Seq.)	Seq.	base	BERT	12	20	640	1024
	**RNABERT**	2022	[50]	0.5M	Human seq. from RNAcentral (~762K Seq.)	Seq.	base	BERT	6	12	120	440
	Uni-RNA (L8)	2023	[27]	21M	RNAcentral and nt [72] and GWH [73] (~1B Seq.)	Seq.	base	BERT	8	8	512	1024
	Uni-RNA (L12)			71M					12	12	768	
	Uni-RNA (L16)			168M					16	16	1280	
	**AIDO.RNA (650 M)**	2024	[42]	648M	RNAcentral (~41.5M Seq.)	Seq.	base	BERT	33	20	1280	1024
	**AIDO.RNA (1.6B)**			1.6B					32	32	2048	
	DGRNA	2024	[55]	108M	MARS (~1.2B) [52]	Seq.	base	Mamba2	12	-	768	2048
	**GenerRNA**	2024	[56]	304M	RNAcentral [19] (~16.09M Seq.)	Seq.	BPE	GPT	24	16	1280	1024
	**RFamLlama (base)**	2024	[57]	31M	Rfam 14.10 (~676K)	Seq.	base	Llama	8	32	512	-
	**RFamLlama (large)**			89M					10	32	768	
	**RNAErnie**	2024	[31]	86M	RNAcentral (~23M Seq.)	Seq.	base	BERT	12	12	768	512
	RNA-km	2024	[32]	152M	RNAcentral (~23M Seq.)	Seq.	base	BERT	12	16	1024	512
	**RNA-MSM**	2024	[29]	96M	MSA (~4000 Rfam [74])	MSA	base	MSA-Transformer	10	12	768	1024
	**BiRNA-BERT**	2025	[43]	116M	RNAcentral and RefSeq [72] (~36.5M Seq.)	Seq.	base or BPE	BERT	12	12	768	1024 (base)
	**ERNIE-RNA**	2025	[28]	86M	RNAcentral (~20.4M Seq.)	Seq.	base	BERT	12	12	768	1024
	**LAMAR (2k)**	2025	[75]	86M	RNAcentral and RefSeq and NCBI Genome (~15M Seq.)	Seq.	base	BERT	12	12	768	2048
	**LAMAR (4k)**											4096
	**ProtRNA**	2025	[40]	651 M	nonredundant RNAcentral (~6M Seq.)	Seq.	base	BERT	33	20	1280	512
	**RiNALMo (micro)**	2025	[30]	33M	RNAcentral and nt and Rfam and Ensembl [76] (~36M Seq.)	Seq.	base	BERT	12	20	480	1024
	**RiNALMo (mega)**			148M					30	20	640	
	**RiNALMo (giga)**			651M					33	20	1280	
II	**3UTRBERT**	2023	[54]	86M	3′UTR in human mRNA transcripts from GENCODE [77] (~20K Seq.)	Seq.	k-mer	BERT	12	12	768	512
	**MP-RNA**	2024	[41]	186M	OneKP initiative [78] (~54.2B Bases)	Seq. and SS	base	BERT	32	30	720	1024
	**PlantRNA-FM**	2024	[53]	34M	OneKP initiative (~54.2B Bases)	Seq. and SS	base	BERT	12	20	480	1026
	**SpliceBERT**	2024	[60]	20M	72 vertebrate genomes from UCSC [79] (~2M Seq.)	Seq.	base	BERT	6	16	512	1024
	**UTR_LM**	2024	[80]	1M	Ensembl-derived 5′UTRs and curated datasets from previous studies [81, 82] (~2.3M Seq.)	Seq. and SS and MFE	base	BERT	6	16	128	1022
III	**LucaOne**	2025	[58]	1.6B	RNA/Refseq (~136M Seq.)	Seq.	base	BERT	20	40	2560	1280

Note: Model size is reported as the number of parameters loaded during inference. For LucaOne, only the number of RNA sequences used for pretraining is reported. ‘Seq.’ denotes RNA sequence; ‘SS’ denotes RNA secondary structure; ‘MFE’ denotes minimum free energy. ‘Max Input Length’ refers to the maximum sequence length or token truncation applied during pretraining and typically inherited during inference.

Despite growing interest in alternative architectures, most of the existing RNA LMs adopt the BERT paradigm (17 out of 21 in Table 1) and rely on masked language modeling as their primary pretraining objective. This architectural dominance mirrors early developments in protein language modeling and reflects the flexibility of encoder-based models for learning contextual sequence representations without task-specific supervision. Consequently, performance differences among RNA LMs arise largely from variations in model scale, training data composition, tokenization schemes, pretraining strategies, and the incorporation of auxiliary inductive biases, rather than from fundamentally distinct network backbones.

**Figure 1:**
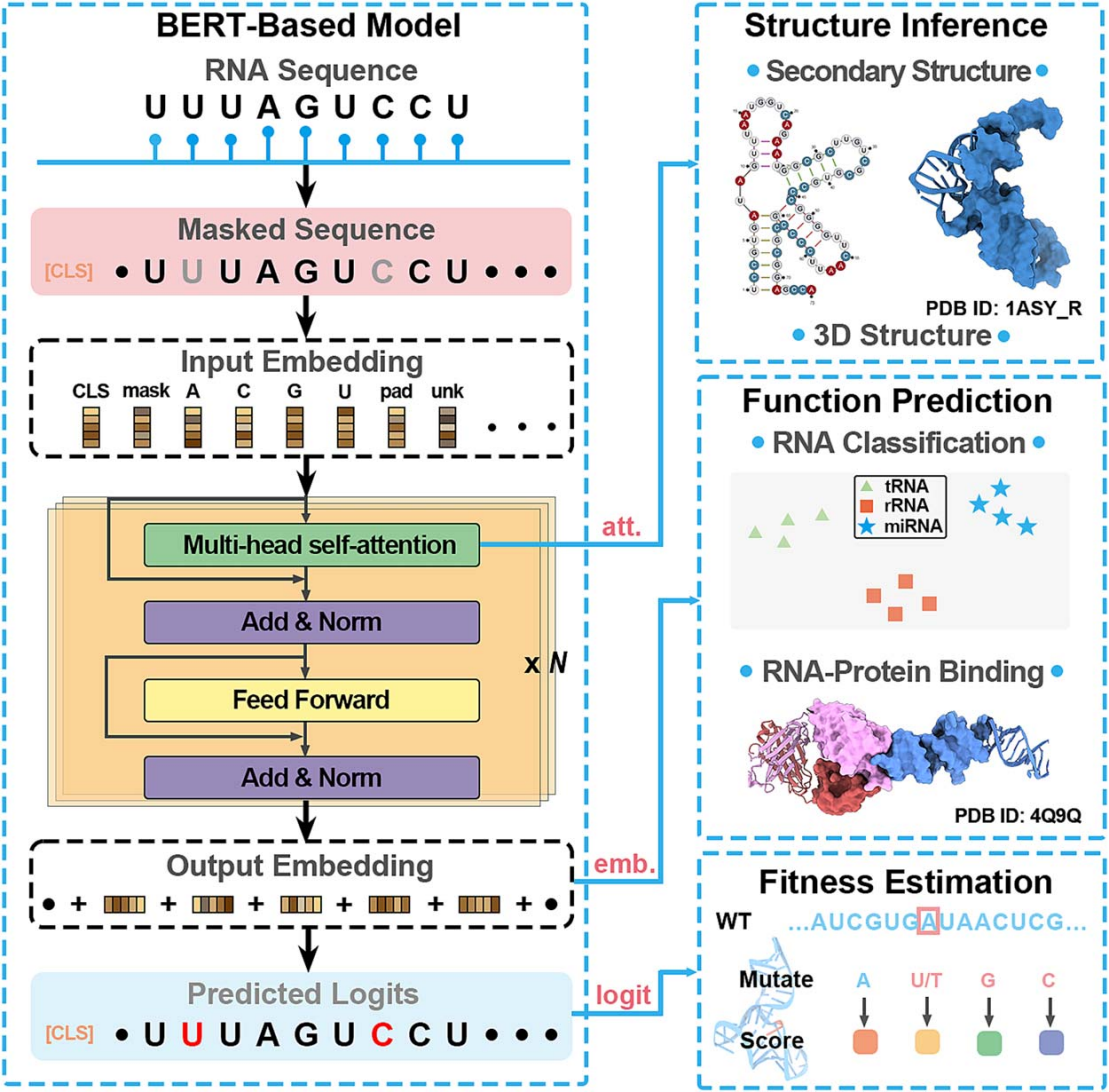
Schematic overview of a BERT-based RNA language model, highlighting three core outputs—attention weights (att.), embedding representations (emb.), and token-level logits (logit). These outputs can be exploited for structural inference, functional prediction, and mutational fitness estimation, respectively.

The canonical network for BERT-based LMs is illustrated in Fig. 1, and a more detailed generalized description is provided in the Supplementary Information. Here, we briefly highlight three model outputs that are particularly relevant for downstream analysis. First, attention weights encode pairwise relationships between sequence positions and have been associated with long-range dependencies and structural organization in biological sequences [26, 30, 35, 37, 51]. Second, last hidden state representations (embeddings) provide contextualized token- or sequence-level representations that form the basis for clustering, classification, and functional or structural analyses [28, 31, 49]. Third, token-level logits, which reflect the model's predicted probability distribution over sequence variants, can be interpreted as proxies for evolutionary constraints and mutational preferences [47, 63]. These three types of outputs—attention weights, embeddings, and logits—are also available in RNA LMs based on alternative architectures, including Mamba2-, GPT-, Llama-, and MSA-Transformer-based models [29, 55–57], and together enable systematic probing of RNA LM behavior in a zero-shot setting.

### Downstream task categories and dataset landscape

Corresponding to the diversity of RNA biological questions, RNA LMs have been evaluated across a wide spectrum of downstream tasks that can be broadly categorized into structural and functional property prediction (Table 2). Structural tasks primarily focus on RNA conformational features and include solvent accessibility prediction [29], secondary structure prediction [26–30, 40–43], three-dimensional (3D) contact and torsion angle prediction [26, 28, 43], and RNA structural alignment [50]. Functional tasks aim to capture regulatory and biological roles of RNA molecules and encompass RNA family or type classification [26, 59], splice sites and modification sites prediction [42, 60], RNA–protein and RNA–RNA interactions prediction [61, 62], mRNA subcellular localizations [54], mean ribosome load (MRL) prediction [26, 30, 40, 42, 80, 81], mRNA expression level (EL) and translation efficiency (TE) prediction [42, 80, 82], genic region annotation [53], internal ribosome entry sites (IRESs) identification [80, 81, 106], RNA generation and mutational fitness prediction [57].

**Table 2:** Summary of downstream tasks used for evaluating RNA LM performance.

**Task**	**Task type**	**Representative LMs**	**Benchmark datasets**
Structural:			
Solvent accessibility	Regression	RNA-MSM	Author-curated solvent accessibility labels computed from RNA 3D chain structures using the POPS package [83] with a probe radius of 1.4 Å [29]
Secondary structure	Binary	RNA-FM, RNA-MSM, Uni-RNA, RiNALMo, ERNIE-RNA, RNAErnie, ProtRNA, MP-RNA, AIDO.RNA	RNAStralign [84]; ArchiveII [85]; bpRNA-1m [86]; RNA 3D structure datasets curated in SPOT-RNA and SPOT-RNA2 [87, 88]
3D contact	Binary	RNA-FM, ERNIE-RNA	RNAcontact benchmark datasets [61], including nonredundant RNA 3D structures curated from Leontis and Zirbel [89]
3D torsion angle	Regression	BiRNA-BERT	RNA 3D structure datasets curated in SPOT-RNA-1d [90]
RNA structure alignment	Alignment/Similarity	RNABERT	RNA pairwise alignments from BRAliBase2.1 k2 [91]
Functional:			
Classification	Binary/Multiclass	RNABERT, BiRNA-BERT, RiNALMo, RNAErnie, LucaOne, AIDO.RNA	ArchiveII [85, 92]; Rfam-based classification benchmarks [74]
Splice sites	Binary	SpliceBERT, RiNALMo, BiRNA-BERT, AIDO.RNA, DGRNA	Author-curated splice-site sequence datasets reported in Spliceator [93]
Modification sites	Multilabel	Uni-RNA, AIDO.RNA, 3UTRBERT	MultiRM benchmark datasets covering 12 RNA modification types [94]; author-curated human m6A modification datasets reported in [54, 95]
RNA-protein binding	Binary	RNA-FM, ERNIE-RNA, ProtRNA, LucaOne, DGRNA, 3UTRBERT	Author-curated RNA–protein interaction datasets reported in PrismNet [62]; ncRPI-LGAT benchmark datasets [96]; author-curated eCLIP-RBP datasets [54]
RNA–RNA binding	Binary	RNAErnie, BiRNA-BERT, DGRNA	Author-curated miRNA-mRNA interactions datasets reported in DeepMirTar [97]; author-curated miRNA-lncRNA interaction datasets reported in PmliPred [98]
mRNA subcellular localizations	Multilabel	3UTRBERT	DM3Loc benchmark datasets [99]; RNA localization datasets curated from RNALocate [100]
Mean ribosome load	Regression	RNA-FM, Uni-RNA, RiNALMo, ERNIE-RNA, UTR_LM, ProtRNA, AIDO.RNA, DGRNA	Author-curated 5′UTR datasets with MRL annotations reported in Optimus 5-prime [81]
mRNA exp. level and trans. efficiency	Regression/Binary	UTR_LM, AIDO.RNA, DGRNA, PlantRNA-FM	Author-curated 5′UTR datasets from human muscle tissue, PC3 prostate cancer cells, and HEK293T cells with corresponding mRNA EL and TE values [80, 82]; author-curated 5′UTR datasets from Arabidopsis and rice transcriptomes with corresponding TE values [53]
RNA genic region annotation	Multilabel	PlantRNA-FM	Author-curated genic region annotation datasets (5′UTR, CDS, and 3′UTR) derived from Phytozome [53, 101]
IRES identification	Binary	UTR_LM	Author-curated IRES identification datasets comprising 46,774 mRNAs collected from multiple public databases [74, 102–105]
RNA generation	Generative/Sequence generation	GenerRNA, RFamLlama	Rfam-based benchmarks [74]
Fitness prediction	Regression	RFamLlama	Author-curated deep mutational scanning (DMS) datasets [57]

Note: Structural and functional tasks are grouped according to their primary evaluation objectives. ‘IRES’ denotes internal ribosome entry sites; ‘RBPs’ denotes RNA-binding proteins. In column ‘Task type’, some regression tasks are binarized in certain studies for evaluation convenience. The column ‘Representative LMs’ lists representative RNA LMs that have been reported to perform the corresponding downstream tasks, rather than an exhaustive enumeration. The column **‘**Benchmark datasets**’** lists commonly used benchmark datasets for each task, where many datasets are author-curated or study-specific datasets without formal names, as originally described in the corresponding references.

Despite their breadth, the use of downstream tasks for evaluating RNA LMs is subject to several fundamental limitations. First, a substantial fraction of evaluations requires task-specific fine-tuning or additional prediction heads, confounding the intrinsic representational capacity of the pretrained models with downstream supervision. Second, data bias and redundancy are prevalent in widely used RNA resources, such as RNAcentral [19] and Rfam [74], where a small number of highly conserved RNA families (e.g. rRNAs and tRNAs) dominate the dataset. Although sequence-level deduplication tools (e.g. MMseqs2 [107] or CD-HIT-EST [108]) are often applied, structurally similar but sequence-divergent RNAs may still lead to information leakage. Third, the quality of evaluation data remains a concern, as many RNA secondary structure benchmarks (e.g. RNAStralign [84], ArchiveII [85, 92], and bpRNA [86]) are derived largely from comparative analysis or predictive algorithms rather than experimentally resolved 3D structures, which is considered the gold standard for revealing all (canonical or noncanonical) base pairs within RNA sequences.

To address these issues, we restrict our analysis to downstream tasks that can be evaluated in a strictly zero-shot setting and that probe complementary aspects of RNA biology. Specifically, RNA secondary structure prediction interrogates structural signals encoded in attention patterns and contextual representations, embedding-based RNA classification evaluates functional and categorical information captured by last hidden state representations, and RNA fitness prediction based on mutational likelihoods leverages token-level logits to quantify probabilistic constraints on sequence variation. In constructing evaluation datasets, we emphasize experimentally derived data wherever possible and explicitly control for data redundancy and imbalance. For secondary structure prediction, we curated a dataset from experimentally resolved PDB structures and applied both sequence- and structure-level redundancy filtering. For RNA classification, positive and negative samples were balanced across RNA families or types. For RNA fitness prediction, we integrated experimental mutational data spanning multiple RNA classes. Together, this evaluation framework aims to provide a more principled, unbiased, and interpretable evaluation of RNA LMs under unified zero-shot conditions.

## Data and methods

### RNA language models included in this study

RNA LMs were collected through systematic searches of peer-reviewed literature and preprint repositories, including Google Scholar, bioRxiv, and arXiv, complemented by citation tracing and curated community resources. Models were included if they (i) were published or publicly available within the past 3 years (before 1 August 2025), (ii) were based on language-model architectures, and (iii) provided open-source pretrained weights enabling direct extraction of model representations.

A total of 21 RNA LMs meeting these criteria were identified, covering general-purpose RNA foundation models, task-specific RNA LMs, and unified cross-molecular LMs. Model characteristics—including pretraining data sources, parameter counts, tokenization strategies, architectural backbones, and details of model availability and download sources—were summarized in Table 1 and Supplementary Table 1. This collection was designed to be representative of the current RNA LM landscape while remaining sufficiently diverse to enable comparative analysis across architectures and training paradigms.

### Datasets

#### RNA secondary structure

For zero-shot evaluation of RNA secondary structure inference, we relied exclusively on base-pair annotations extracted from experimentally resolved 3D RNA structures, as high-resolution 3D data provide the most reliable ground truth at the base-pair level. We adopted a previously curated dataset comprising a validation set (VL1, 40 PDBs) and an independent test set (TS, 70 PDBs) [29]. Redundancy was removed at both sequence and structural levels, with sequence identity capped below 80% and structural similarity (TM-score) below 0.45 between validation and test sets. In addition, overlap in Rfam family annotations between VL1 and TS was eliminated using Infernal-based homology detection [109]. This resulted in a strictly nonhomologous subset (TS-Hard, 15 PDBs), which was used to assess robustness under stringent homology exclusion.

#### RNA classification

Functional similarity was evaluated using two complementary RNA classification benchmarks that operate at different levels of granularity. For RNA family–level classification, we constructed the RfamSample dataset based on Rfam 14.0 seed alignments [74]. After internal deduplication within each family (sequence identity below 80%), 10 sequences (length ≤ 1024) were randomly sampled from each family containing more than 10 members, yielding a total of 561 families. This dataset was used to examine the ability of RNA LMs to distinguish within-family from cross-family sequence pairs. For RNA type–level classification, we employed the ArchiveII [85] dataset after excluding excessively long group II introns. To reduce redundancy, MMseqs2 [107] was applied with a maximum sequence identity threshold of 80%, resulting in a nonredundant subset (ArchiveII-Nr) containing 1273 RNAs (Supplementary Table 2). This dataset was chosen to probe whether RNA LM embeddings can capture functional similarity beyond close sequence homology.

#### RNA DMS assays

Zero-shot RNA fitness prediction was evaluated using experimental mutational datasets from the RNAGym benchmark [63]. We selected 31 ncRNA deep mutational scanning (DMS) assays with wild-type sequence lengths not exceeding 440 nucleotides, ensuring compatibility with all evaluated LMs. These assays span multiple RNA classes and provide quantitative measurements of mutational effects suitable for logit-based evaluation.

### Zero-shot evaluation

All evaluations were conducted in a strictly zero-shot setting, without any task-specific fine-tuning or additional supervised training.

#### Zero-shot secondary structure prediction 

For each RNA LM, attention maps were extracted from all head–layer combinations. Attention matrices were symmetrized, corrected using average product correction (APC), and transformed into base-pairing probability matrices via sigmoid scaling. A single optimal head–layer and decision threshold was selected on the validation set (VL1) by maximizing the F1 score and then applied unchanged to the test sets (TS and TS-Hard). Model performance was quantified using Matthews correlation coefficient (MCC) and F1 score. This procedure ensures that performance reflects intrinsic structural information encoded during pretraining rather than after task-specific optimization.

#### Zero-shot RNA classification by embedding similarity

To assess whether sequence embeddings encode functional similarity, sequence embeddings were extracted and converted into fixed-length representations using Fourier transform-based dimensionality reduction (FFT). Cosine similarity distributions were computed for homologous and nonhomologous RNA sequence pairs sampled from RfamSample and ArchiveII-Nr datasets. Model performance was quantified using (i) MCC, (ii) area under the receiver operating characteristic curve (AUC), and (iii) the overlap ratio (OR) between similarity distributions, with lower OR indicating stronger discriminative capacity. In addition to FFT-based embeddings, the dedicated classification token (CLS) embedding was employed as a complementary representation to assess method dependence [30].

#### Zero-shot RNA fitness prediction via mutational likelihoods

RNA fitness prediction was performed using token-level logits to estimate the likelihood of sequence variants, following established zero-shot evaluation protocols [47, 63]. Two scoring schemes were considered: wild-type conditioned log-likelihood ratios (WT-LLR) and pseudo-log-likelihood differences (PLL-D), capturing local and global mutational effects, respectively. For autoregressive models and k-mer/BPE–based models, only PLL-D were computed. Logit normalization was performed using either log-softmax or softmax transformations, depending on model output characteristics, to ensure comparability across different models. Model performance was quantified using (i) MCC, (ii) AUC, and (iii) the absolute value of the Spearman correlation coefficient (SR) between predicted and experimental mutational effects.

### Statistical analysis

All reported results were obtained without downstream fine-tuning. Performance metrics represent averages over multiple independent random samplings where applicable. Supplementary Table 3 summarizes the availability of attention weights, embedding representations, and token-level logits across evaluated RNA and DNA LMs, which determined the inclusion of specific LMs in each zero-shot task.

Additional methodological details are provided in the Supplementary Information.

## Results

### Structural signals encoded by attention: zero-shot RNA secondary structure prediction

We first examined whether RNA LMs capture RNA secondary structure information through attention-based analysis. Following prior work [29], attention maps extracted from RNA LMs were used to perform zero-shot RNA secondary structure prediction. A single optimal head–layer combination and decision threshold were selected on a validation set (VL1) and subsequently applied unchanged to an independent test set (TS), which consists of 70 RNAs with experimentally determined 3D structures. The DNA LM DNABERT [38] was included as a reference control.

**Figure 2:**
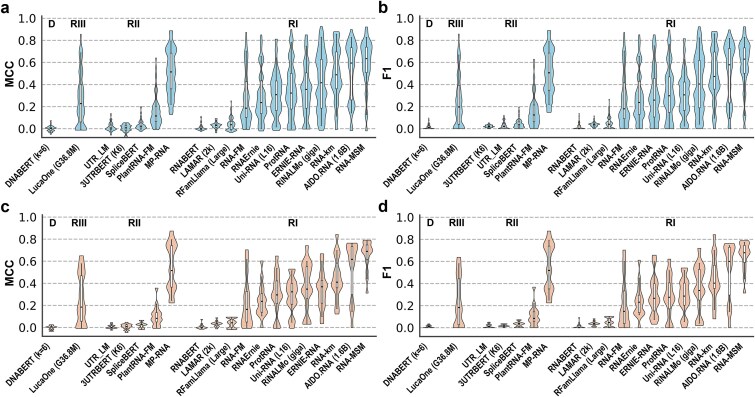
Zero-shot RNA secondary structure prediction by DNA LMs (D), Class III (unified, RIII), II (mRNA-related, RII), and I (ncRNA-enriched, RI) RNA LMs. Performance was evaluated using (a) MCC and (b) F1 score on the test set (TS), and (c) MCC and (d) F1 score on the hard test set (TS-Hard). For models with multiple released versions, the version achieving the highest median MCC was shown. Models within each category were ordered by increasing median MCC.

As summarized in Supplementary Table 4 and Fig. 2a and b, performance evaluated by MCC and F1 score revealed that the DNABERT and several Class II RNA LMs trained primarily on mRNA-related sequences—including 3UTRBERT, SpliceBERT, and UTR_LM—exhibited negligible secondary structure prediction capability, underscoring the importance of ncRNA-rich pretraining corpora for capturing RNA structural information. Notable exceptions were MP-RNA and PlantRNA-FM, which achieved relatively strong performance despite limited ncRNA content in their training data. Their effectiveness can be attributed to the explicit incorporation of RNA secondary structure information, derived from ViennaRNA-based predictions, during pretraining and inference [41, 53, 110]. The unified multiomics model LucaOne (Class III RNA LM) showed moderate performance but consistently underperformed most Class I RNA LMs, suggesting that modality-agnostic pretraining may dilute RNA-specific structural inductive biases.

Among Class I RNA LMs, RNA-MSM achieved the highest median F1 score and MCC, outperforming AIDO.RNA (1.6B) by 9.0% and 6.6%, respectively, despite being trained on fewer than 4000 RNA families. This result highlights the advantage of incorporating evolutionary information via multiple sequence alignments and emphasizes the intrinsic difficulty of RNA LMs for inferring RNA secondary structure from single sequences alone. Clear scaling effects were observed across several model families: larger models consistently outperformed their smaller counterparts, including RiNALMo (giga versus mega), Uni-RNA (L16 versus L12), and AIDO.RNA (1.6B versus 650M).

Essentially the same ranking was observed on the more stringent TS-Hard dataset, which shares no RNA families with the validation set (VL1). The Spearman correlation coefficient between median MCC values on TS and TS-Hard was 0.99, indicating strong ranking consistency. As shown in Supplementary Table 5 and Fig. 2c and d, the top-performing models on TS—MP-RNA (#3), AIDO.RNA (1.6B) (#2), and RNA-MSM (#1)—maintained their relative ordering on TS-Hard, supporting the robustness of the comparative analysis despite the limited size of the hard test set.

### Functional signals in embedding representations: zero-shot RNA classification

To evaluate whether RNA LM embeddings encode functional and categorical information, we performed zero-shot RNA classification based on embedding similarity. A total of 100,000 homologous and 100,000 nonhomologous RNA sequence pairs were sampled from the RfamSample dataset, and cosine similarity between sequence-level embeddings was computed using a Fourier transform–based dimensionality reduction approach (details in Methods). DNA LMs (e.g. DNABERT [38], DNABERT-2 [39], and NT [33]) were included as controls. Model performance was quantified using MCC, AUC, and the OR. For consistency with MCC and AUC, the 1 – OR was reported, such that higher values indicate better performance.

**Figure 3:**
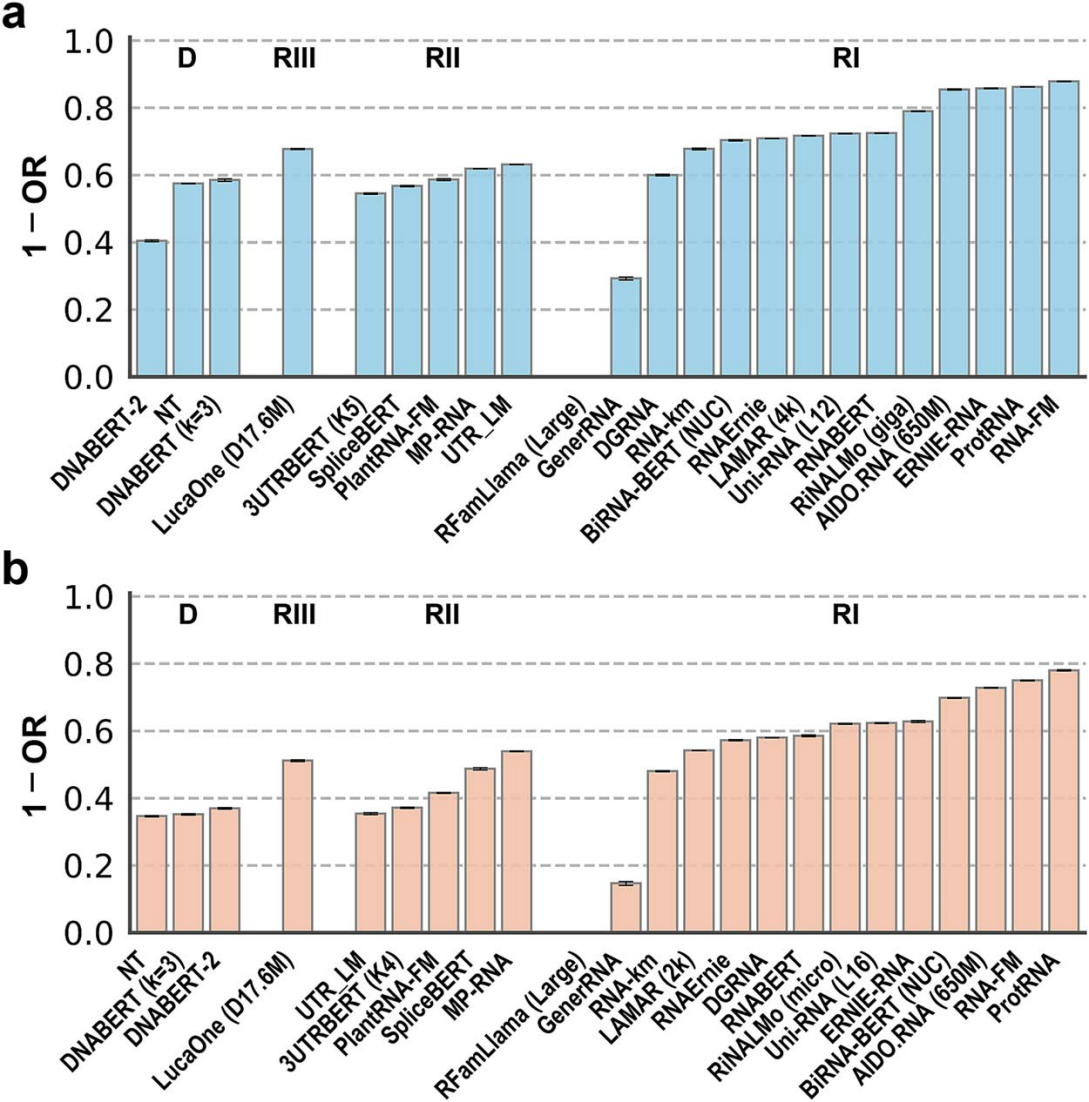
Zero-shot RNA classification based on embedding similarity on (a) the RfamSample and (b) ArchiveII-Nr datasets. Results were shown for DNA LMs (D), Class III (unified, RIII), II (mRNA-related, RII), and I (ncRNA-enriched, RI) RNA LMs. Performance was measured by the OR between similarity distributions, reported as 1 – OR (better performance for a larger value). For the models with multiple versions, the version with the highest mean 1 − OR was displayed. Models within each category were ordered by increasing mean 1 − OR.

As shown in Supplementary Table 6 and Fig. 3a, most RNA and DNA LMs were able to distinguish homologous from nonhomologous sequence pairs, indicating that unsupervised pretraining captures family-level RNA properties to some extent. Overall, Class I RNA LMs outperformed DNA LMs and Class II RNA LMs on the RfamSample dataset, whereas the unified model LucaOne showed mid-range performance. RNA-FM achieved the strongest discriminative performance, with a mean value of 1 − OR = 0.878 ± 0.001. This was followed by ProtRNA. In contrast, non-BERT-based models (e.g. DGRNA, GenerRNA, and RFamLlama) produced embeddings with limited discriminative power in the absence of task-specific prompts.

Classification results based on F1, MCC, and AUC (Supplementary Table 6) were highly consistent with those based on 1 − OR, with Spearman correlation coefficients of 0.95, 0.94, and 0.89, respectively. This consistency indicates that the OR provides a robust summary metric for embedding-based classification performance.

We further evaluated model performance on the ArchiveII-Nr dataset, where homology was defined at the RNA type level rather than the family level. As shown in Supplementary Table 7 and Fig. 3b, overall performance decreased across all models, reflecting increased sequence diversity and weaker homology signals. Nevertheless, model rankings remained largely consistent with those observed on RfamSample (Spearman correlation = 0.88). ProtRNA was now the best, followed by RNA-FM. Notably, increased model size did not systematically improve performance in this task; in several cases, smaller models outperformed larger ones, suggesting that embedding quality does not scale monotonically with parameter count.

Complementary analyses using the dedicated classification token (CLS) embedding [30] yielded highly similar results (Supplementary Tables 8 and 9). The Spearman correlation between FFT-based and CLS-based rankings reached up to 0.95 for mean 1 − OR on both datasets, further supporting the robustness of the observed trends. In addition, high correlations in model rankings were also observed between 1 − OR and MCC (Spearman correlation = 0.92 in RfamSample and 0.94 in ArchiveII-Nr), as well as between 1 − OR and AUC (Spearman correlation = 0.93 in RfamSample and 0.94 in ArchiveII-Nr), when classification performance was evaluated using CLS-based embeddings.

### Probabilistic signals in token-level logits: zero-shot RNA fitness prediction

Finally, we examined whether RNA LMs encode evolutionary and functional constraints through token-level logits by evaluating zero-shot RNA fitness prediction. Experimental mutational assays from the RNAGym benchmark [63], covering multiple classes of ncRNAs, were used for evaluation, with DNA LMs serving as reference controls. Model performance was quantified using MCC, AUC, and the absolute value of the Spearman correlation coefficient (SR).

Consistent with observations in ProteinGym [47] and RNAGym [63], the choice of scoring function had a pronounced impact on performance. As shown in Supplementary Tables 10 and 11, WT-LLRs substantially outperformed PLL-Ds across most RNA LMs and were used by nearly all top-performing models. This result highlights the importance of wild-type–anchored scoring for zero-shot RNA mutation effect prediction.

**Figure 4:**
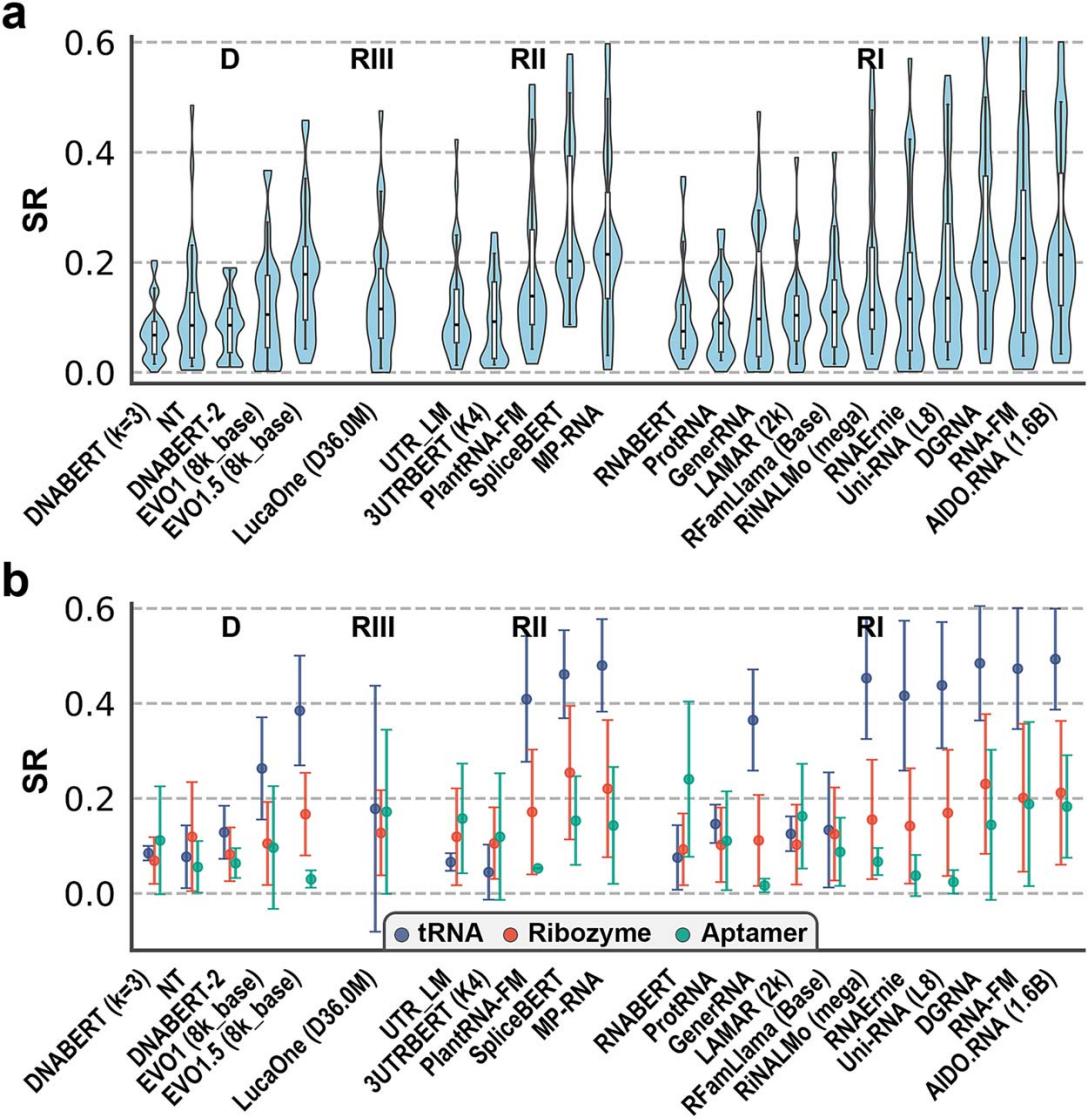
Zero-shot RNA fitness prediction using token-level logits. (a) Overall performance of DNA LMs (D), Class III (unified, RIII), II (mRNA-related, RII), and I (ncRNA-enriched, RI) RNA LMs, measured by the absolute value of the Spearman correlation coefficient (SR) between predicted and experimental mutational effects. For models with multiple versions, only the version with the highest median SR was shown. Models within each category were ordered by increasing median SR. (b) Median SR across assays grouped by RNA type, illustrating RNA class–dependent performance variability.

Overall performance patterns differed markedly from those observed in structural and classification tasks. As summarized in Fig. 4a (best results for each LM, using either the WT-LLR or PLL-D method), DNA LMs exhibited limited predictive power across all metrics. Models incorporating explicit evolutionary modeling or extended context lengths (e.g. EVO1.5) showed modest improvements but still underperformed RNA-specialized models. Unified models (LucaOne family) consistently outperformed DNA LMs, except for the EVO series, but remained inferior to many RNA-focused models.

The best performance was achieved by several Class I and Class II RNA LMs, including MP-RNA, AIDO.RNA, RNA-FM, SpliceBERT, and DGRNA, with median MCC values of 0.14–0.18, median AUC of approximately 0.60, and median SR values exceeding 0.20. MP-RNA and SpliceBERT show the strongest performance based on median (0.215) and mean (0.268) SR values, respectively. Nevertheless, absolute performance remained limited across all models (mean MCC < 0.20, AUC < 0.64, SR < 0.27), indicating that zero-shot RNA fitness prediction remains a challenging task. Model rankings based on MCC, AUC, and SR were highly consistent, with Spearman correlations of 0.99 between SR and the other metrics at the mean level.

Despite low overall performance, prediction accuracy varied substantially across RNA types. As shown in Fig. 4b, models with strong overall performance (e.g. SpliceBERT, DGRNA, EVO1.5, etc.) derived much of their predictive power from tRNA assays, where SR values exceeded 0.4, whereas performance on other RNA classes remained comparatively weak.

## Discussion

Over the past decade, interest in RNA biology—particularly ncRNAs—has expanded rapidly due to their diverse regulatory and functional roles [1, 2, 8, 9]. Despite the central role of structure in determining RNA function, experimentally resolved RNA structures account for less than 0.1% of known sequences [111]. Inspired by the success of PLMs in capturing structure–function relationships from sequence alone, RNA LMs have emerged as a promising framework for large-scale RNA analysis [26, 27, 29, 30]. Here, we performed unified zero-shot evaluations of 21 RNA LMs to assess how effectively they encode structural, functional, and evolutionary information.

Consistent with prior observations, the limited nucleotide alphabet and weak sequence conservation of RNAs make structure inference from single sequences intrinsically challenging [26, 29, 112]. This difficulty is underscored by the strong performance of the MSA-based RNA-MSM model, which outperforms single-sequence RNA LMs despite being pretrained on a comparatively small number of RNA families. Although model scaling contributes to improved performance, it is not clear if simply expanding the number of parameters in existing RNA LMs would resolve the performance gap between MSA-based and single-sequence-based LMs.

A key finding of this study is the pronounced trade-off among structural, functional, and evolutionary representations. Models that perform well in zero-shot secondary structure prediction (e.g. RNA-km and MP-RNA) may exhibit weaker performance in embedding-based RNA classification or logit-based fitness prediction and vice versa. These results indicate that different biological signals are not automatically aligned in current RNA LMs and that pretraining strategies favoring one aspect may compromise others.

We further observe that unified or multiomics LMs do not consistently outperform RNA-specialized models on RNA-centric tasks. While such models may be advantageous for cross-modal applications, RNA-specific inductive biases appear critical for accurately capturing RNA structural and functional properties.

Together, these findings highlight substantial opportunities for improving RNA LMs. Although continued scaling is likely to yield incremental gains, more substantial progress will require advances in training objectives, data design, and inductive bias integration. Improving structural representations without degrading functional and evolutionary signals remains a central challenge. One possible solution is to develop domain-specific LMs that may offer some advantages for specific applications. Moreover, most current models treat RNA as composed solely of four canonical nucleotides, despite the widespread presence of chemical modifications that influence RNA folding and function. Incorporating RNA modification information therefore represents an important direction for future model development.

In summary, our results demonstrate that existing RNA LMs encode heterogeneous and task-dependent biological signals. A clearer understanding of how model architecture, training data, and objectives shape structural, functional, and evolutionary representations will be essential for developing next-generation RNA LMs that more faithfully reflect all aspects of RNA biology.

Key Points

- RNA language models are evaluated using a strictly zero-shot framework, enabling assessment of intrinsic representational capacity independent of downstream fine-tuning.
- Three zero-shot tasks—secondary structure inference, RNA classification, and mutational fitness prediction—are selected to explicitly correspond to attention weights, embeddings, and token-level logits, respectively.
- Current RNA language models encode heterogeneous and task-dependent biological signals, with clear trade-offs between structural, functional, and evolutionary representations.
- This work provides a standardized benchmark and evaluation strategy to facilitate more interpretable and reproducible comparisons of RNA language models.

## Supplementary Material

bbag098_Supplymentary_Information_20260209

## Data Availability

Datasets for zero-shot evaluations are available at https://zenodo.org/records/18218776.

## References

[1] Chaudhary N, Weissman D, Whitehead KA. mRNA vaccines for infectious diseases: principles, delivery and clinical translation. *Nat Rev Drug Discov* 2021;20:817–38. 10.1038/s41573-021-00283-5. 34433919 PMC8386155

[2] Rohner E, Yang R, Foo KS. et al. Unlocking the promise of mRNA therapeutics. *Nat Biotechnol* 2022;40:1586–600. 10.1038/s41587-022-01491-z. 36329321

[3] Curreri A, Sankholkar D, Mitragotri S. et al. RNA therapeutics in the clinic. *Bioeng Transl Med* 2023;8:e10374. 10.1002/btm2.10374. 36684099 PMC9842029

[4] Gayet RV, Ilia K, Razavi S. et al. Autocatalytic base editing for RNA-responsive translational control. *Nat Commun* 2023;14:1339. 10.1038/s41467-023-36851-z. 36906659 PMC10008589

[5] Dykstra PB, Kaplan M, Smolke CD. Engineering synthetic RNA devices for cell control. *Nat Rev Genet* 2022;23:215–28. 10.1038/s41576-021-00436-7. 34983970 PMC9554294

[6] Li B, Niu Y, Ji W. et al. Strategies for the CRISPR-based therapeutics. *Trends Pharmacol Sci* 2020;41:55–65. 10.1016/j.tips.2019.11.006. 31862124 PMC10082448

[7] Bora RS, Gupta D, Mukkur TKS. et al. RNA interference therapeutics for cancer: challenges and opportunities (review). *Mol Med Rep* 2012;6:9–15. 10.3892/mmr.2012.871. 22576734

[8] Childs-Disney JL, Yang X, Gibaut QMR. et al. Targeting RNA structures with small molecules. *Nat Rev Drug Discov* 2022;21:736–62. 10.1038/s41573-022-00521-4. 35941229 PMC9360655

[9] Garner AL . Contemporary progress and opportunities in RNA-targeted drug discovery. *ACS Med Chem Lett* 2023;14:251–9. 36923915 10.1021/acsmedchemlett.3c00020 PMC10009794

[10] Morris KV, Mattick JS. The rise of regulatory RNA. *Nat Rev Genet* 2014;15:423–37. 10.1038/nrg3722. 24776770 PMC4314111

[11] Bohnsack MT, Sloan KE. Modifications in small nuclear RNAs and their roles in spliceosome assembly and function. *Biol Chem* 2018;399:1265–76. 10.1515/hsz-2018-0205. 29908124

[12] Caprara MG, Nilsen TW. RNA: versatility in form and function. *Nat Struct Biol* 2000;7:831–3. 11017186 10.1038/82816

[13] Sharp PA . The centrality of RNA. *Cell* 2009;136:577–80. 10.1016/j.cell.2009.02.007. 19239877

[14] Fu X-D . Non-coding RNA: a new frontier in regulatory biology. *Natl Sci Rev* 2014;1:190–204. 25821635 10.1093/nsr/nwu008 PMC4374487

[15] Mattick JS, Makunin IV. Non-coding RNA. *Hum Mol Genet* 2006;15:R17–29. 10.1093/hmg/ddl046. 16651366

[16] Wang KC, Chang HY. Molecular mechanisms of long noncoding RNAs. *Mol Cell* 2011;43:904–14. 10.1016/j.molcel.2011.08.018. 21925379 PMC3199020

[17] Bartel DP . MicroRNAs: genomics, biogenesis, mechanism, and function. *Cell* 2004;116:281–97. 14744438 10.1016/s0092-8674(04)00045-5

[18] Kung JTY, Colognori D, Lee JT. Long noncoding RNAs: past, present, and future. *Genetics* 2013;193:651–69. 10.1534/genetics.112.146704. 23463798 PMC3583990

[19] RNAcentral Consortium . RNAcentral 2021: secondary structure integration, improved sequence search and new member databases. *Nucleic Acids Res* 2021;49:D212–20. 33106848 10.1093/nar/gkaa921 PMC7779037

[20] Hong X, Zhan J, Zhou Y. On the completeness of existing RNA fragment structures. *Genomics Proteomics Bioinformatics* 2025;23:qzaf127. 10.1093/gpbjnl/qzaf127. PMC13197129 41414643

[21] Vaswani A, Shazeer N, Parmar N. et al. Attention is all you need. In: *Proceedings of the 31st International Conference on Neural Information Processing Systems 6000–6010*. Long Beach, California, USA: Curran Associates Inc., 2017.

[22] Devlin J, Chang M-W, Lee K. et al. BERT: Pre-training of deep bidirectional transformers for language understanding. In: Burstein J, Doran C, Solorio T, (eds.), Proceedings of the 2019 Conference of the North American Chapter of the Association for Computational Linguistics: Human Language Technologies, Volume 1 (Long and Short Papers). Minneapolis, Minnesota: Association for Computational Linguistics, 2019, 4171–86.

[23] Radford A, Narasimhan K. Improving Language Understanding by Generative Pre-Training. https://api.semanticscholar.org/CorpusID:49313245, 2018.

[24] Brown T, Mann B, Ryder N. Language models are few-shot learners. In: Larochelle H, Ranzato M, Hadsell R. et al., (eds.), Vol. 33 Advances in Neural Information Processing Systems. New York: Curran Associates, Inc., 2020, 1877–901.

[25] Raffel C, Shazeer N, Roberts A. et al. Exploring the limits of transfer learning with a unified text-to-text transformer. *J Mach Learn Res* 2020;21:1–67.34305477

[26] Chen J, Hu Z, Sun S. et al. Interpretable RNA foundation model from unannotated data for highly accurate RNA structure and function predictions. *arXiv e-prints* arXiv:2204.00300. 2022. 10.48550/arXiv.2204.00300.

[27] Wang X, Gu R, Chen Z. et al. Uni-RNA: universal pre-trained models revolutionize RNA RESEARCH. *bioRxiv* 2023.07.11.548588. 2023. 10.1101/2023.07.11.548588.

[28] Yin W, Zhang Z, Zhang S. et al. ERNIE-RNA: an RNA language model with structure-enhanced representations. *Nat Commun* 2025;16:10076. 10.1038/s41467-025-64972-0. 41253752 PMC12627772

[29] Zhang Y, Lang M, Jiang J. et al. Multiple sequence alignment-based RNA language model and its application to structural inference. *Nucleic Acids Res* 2024;52:e3–3. 37941140 10.1093/nar/gkad1031 PMC10783488

[30] Penić RJ, Vlašić T, Huber RG. et al. RiNALMo: general-purpose RNA language models can generalize well on structure prediction tasks. *Nat Commun* 2025;16:5671. 10.1038/s41467-025-60872-5. 40593636 PMC12219582

[31] Wang N, Bian J, Li Y. et al. Multi-purpose RNA language modelling with motif-aware pretraining and type-guided fine-tuning. *Nat Mach Intell* 2024;6:548–57.

[32] Gong T, Bu D. Language models enable zero-shot prediction of RNA secondary structure including pseudoknots. *bioRxiv* 2024.01.27.577533. 2024. 10.1101/2024.01.27.577533. PMC10924946 38461362

[33] Dalla-Torre H, Gonzalez L, Mendoza-Revilla J. et al. Nucleotide transformer: building and evaluating robust foundation models for human genomics. *Nat Methods* 2025;22:287–97. 10.1038/s41592-024-02523-z. 39609566 PMC11810778

[34] Nijkamp E, Ruffolo JA, Weinstein EN. et al. ProGen2: exploring the boundaries of protein language models. *Cell Syst* 2023;14:968–978.e3. 10.1016/j.cels.2023.10.002. 37909046

[35] Rives A, Meier J, Sercu T. et al. Biological structure and function emerge from scaling unsupervised learning to 250 million protein sequences. *Proc Natl Acad Sci U S A* 2021;118:e2016239118. 10.1073/pnas.2016239118. PMC8053943 33876751

[36] Rao R, Liu J, Verkuil R. et al. MSA transformer. bioRxiv. 2021. 10.1101/2021.02.12.430858.

[37] Elnaggar A, Heinzinger M, Dallago C. et al. ProtTrans: toward understanding the language of life through self-supervised learning. *IEEE Trans Pattern Anal Mach Intell* 2022;44:7112–27. 10.1109/TPAMI.2021.3095381. 34232869

[38] Ji Y, Zhou Z, Liu H. et al. DNABERT: pre-trained bidirectional encoder representations from transformers model for DNA-language in genome. *Bioinformatics* 2021;37:2112–20. 10.1093/bioinformatics/btab083. 33538820 PMC11025658

[39] Zhou Z, Ji Y, Li W. et al. DNABERT-2: Efficient foundation model and benchmark for multi-species genomes. In: Kim B, (ed.), et al. International Conference on Representation Learning, 2024, 41642–65. Vol. 2024

[40] Zhang R, Ma B, Xu G. et al. ProtRNA: a protein-derived RNA language model by cross-modality transfer learning. *Cell Syst* 2025;16:101371. 10.1016/j.cels.2025.101371. 40848715

[41] Yang H, Li K. MP-RNA: Unleashing multi-species RNA foundation model via calibrated secondary structure prediction. In: Al-Onaizan Y, Bansal M, Chen Y-N, (eds.), Findings of the Association for Computational Linguistics: EMNLP 2024. Miami, Florida, USA: Association for Computational Linguistics, 2024, 5278–96.

[42] Zou S, Tao T, Mahbub S. et al. A large-scale foundation model for RNA function and structure prediction. In: NeurIPS 2024 Workshop on AI for New Drug Modalities, 2024.

[43] Tahmid MT, Shahgir HS, Mahbub S. et al. BiRNA-BERT allows efficient RNA language modeling with adaptive tokenization. *Commun Biol* 2025;8:1621. 10.1038/s42003-025-08982-0. 41266599 PMC12635123

[44] Zhou H, Yin M, Wu W. et al. ProtCLIP: function-informed protein multi-modal learning. *AAAI* 2025;39:22937–45.

[45] Xiao Y, Sun E, Jin Y. et al. ProteinGPT: Multimodal LLM for protein property prediction and structure understanding. In: ICLR 2025 Workshop on Machine Learning for Genomics Explorations, 2025.

[46] Hayes T, Rao R, Akin H. et al. Simulating 500 million years of evolution with a language model. *Science* 2025;387:850–8. 10.1126/science.ads0018. 39818825

[47] Notin P, Kollasch A, Ritter D. et al. ProteinGym: Large-scale benchmarks for protein fitness prediction and design. In: Proceedings of the 37th International Conference on Neural Information Processing Systems. New Orleans, LA, USA: Curran Associates Inc., 2023.

[48] Xu M, Zhang Z, Lu J. et al. PEER: a comprehensive and multi-task benchmark for protein sequence understanding. In: Koyejo S, (ed.), et al. Advances in Neural Information Processing Systems. Curran Associates, Inc., 2022, 35156–73. Vol. 35

[49] Chen J-Y, Wang JF, Hu Y. et al. Evaluating the advancements in protein language models for encoding strategies in protein function prediction: a comprehensive review. *Front Bioeng Biotechnol* 2025;13:1506508. 39906415 10.3389/fbioe.2025.1506508 PMC11790633

[50] Akiyama M, Sakakibara Y. Informative RNA base embedding for RNA structural alignment and clustering by deep representation learning. *NAR Genom Bioinform* 2022;4:lqac012. 10.1093/nargab/lqac012. 35211670 PMC8862729

[51] Shen T, Hu Z, Sun S. et al. Accurate RNA 3D structure prediction using a language model-based deep learning approach. *Nat Methods* 2024;21:2287–98. 10.1038/s41592-024-02487-0. 39572716 PMC11621015

[52] Chen K, Litfin T, Singh J. et al. MARS and RNAcmap3: the master database of all possible RNA sequences integrated with RNAcmap for RNA homology search. *Genomics Proteomics Bioinformatics* 2024;22:qzae018. 10.1093/gpbjnl/qzae018. 38872612 PMC12053375

[53] Yu H, Yang H, Sun W. et al. An interpretable RNA foundation model for exploring functional RNA motifs in plants. *Nat Mach Intell* 2024;6:1616–25. 10.1038/s42256-024-00946-z. 39703563 PMC11652376

[54] Yang Y, Li G, Pang K. et al. Deciphering 3’UTR mediated gene regulation using interpretable deep representation learning. *Adv Sci* 2024;11:2407013. 10.1002/advs.202407013. PMC11497048 39159140

[55] Yuan Y, Chen Q, Pan X. DGRNA: a long-context RNA foundation model with bidirectional attention Mamba2. *bioRxiv* 2024.10.31.621427. 2024. 10.1101/2024.10.31.621427.

[56] Zhao Y, Oono K, Takizawa H. et al. GenerRNA: A generative pre-trained language model for de novo RNA design. *PloS One* 2024;19:e0310814. 10.1371/journal.pone.0310814. 39352899 PMC11444397

[57] Sun J, Li H, Deng Y. RFamLlama: An efficient conditional language model for RNA sequence generation across diverse structural families. In: ICML 2024 Workshop on Efficient and Accessible Foundation Models for Biological Discovery, 2024.

[58] He Y, Fang P, Shan Y. et al. Generalized biological foundation model with unified nucleic acid and protein language. *Nat Mach Intell* 2025;7:942–53.

[59] Rossi E, Monti F, Bronstein M. et al. ncRNA classification with graph convolutional networks. *arXiv e-prints* arXiv:1905.06515. 2019. 10.48550/arXiv.1905.06515.

[60] Chen K, Zhou Y, Ding M. et al. Self-supervised learning on millions of primary RNA sequences from 72 vertebrates improves sequence-based RNA splicing prediction. *Brief Bioinform* 2024;25:bbae163. 10.1093/bib/bbae163. 38605640 PMC11009468

[61] Sun S, Wang W, Peng Z. et al. RNA inter-nucleotide 3D closeness prediction by deep residual neural networks. *Bioinformatics* 2021;37:1093–8. 33135062 10.1093/bioinformatics/btaa932 PMC8150135

[62] Xu Y, Zhu J, Huang W. et al. PrismNet: predicting protein-RNA interaction using in vivo RNA structural information. *Nucleic Acids Res* 2023;51:W468–77. 10.1093/nar/gkad353. 37140045 PMC10320048

[63] Arora R, Angelo M, Choe CA. et al. RNAGym: Benchmarks for RNA fitness and structure prediction. In: ICLR 2025 Workshop on AI for Nucleic Acids, 2025.

[64] Wang H, Lin W, Xiao H. et al. RNA-scope: Benchmarking RNA language models for RNA sequence understanding. In: NeurIPS 2025 AI for Science Workshop, 2025.

[65] Jumper J, Evans R, Pritzel A. et al. Highly accurate protein structure prediction with AlphaFold. *Nature* 2021;596:583–9. 10.1038/s41586-021-03819-2. 34265844 PMC8371605

[66] Lin Z, Akin H, Rao R. et al. Evolutionary-scale prediction of atomic-level protein structure with a language model. *Science* 2023;379:1123–30. 10.1126/science.ade2574. 36927031

[67] Wu R, Ding F, Wang R. et al. High-resolution de novo structure prediction from primary sequence. *bioRxiv* 2022.07.21.500999. 2022. 10.1101/2022.07.21.500999.

[68] Kretsch RC, Hummer AM, He S. et al. Assessment of nucleic acid structure prediction in CASP16. *Proteins* 2026;94:192–217. 10.1002/prot.70072. 41165252 PMC13185081

[69] Zablocki LI, Bugnon LA, Gerard M. et al. Comprehensive benchmarking of large language models for RNA secondary structure prediction. *Brief Bioinform* 2025;26:bbaf137. 10.1093/bib/bbaf137. 40205851 PMC11982019

[70] Li Z, Ma R, Tan J. et al. NABench: large-scale benchmarks of nucleotide foundation models for fitness prediction. *arXiv e-prints* arXiv:2511.02888. 2025. 10.48550/arXiv.2511.02888.

[71] Ren Y, Chen Z, Qiao L. et al. BEACON: Benchmark for comprehensive RNA tasks and language models. In: Proceedings of the 38th International Conference on Neural Information Processing Systems. Vancouver, BC, Canada: Curran Associates Inc., 2024.

[72] Sayers EW, Bolton EE, Brister JR. et al. Database resources of the National Center for biotechnology information in 2023. *Nucleic Acids Res* 2023;51:D29–38. 10.1093/nar/gkac1032. 36370100 PMC9825438

[73] Chen M, Ma Y, Wu S. et al. Genome warehouse: a public repository housing genome-scale data. *Genomics Proteomics Bioinformatics* 2021;19:584–9. 10.1016/j.gpb.2021.04.001. 34175476 PMC9039550

[74] Kalvari I, Nawrocki EP, Ontiveros-Palacios N. et al. Rfam 14: expanded coverage of metagenomic, viral and microRNA families. *Nucleic Acids Res* 2021;49:D192–200. 10.1093/nar/gkaa1047. 33211869 PMC7779021

[75] Zhou H, Hu Y, Zheng Y. et al. A foundation language model to decipher diverse regulation of RNAs. *Genome Biol* 2025;26:301. 10.1186/s13059-025-03752-x. 40993693 PMC12462214

[76] Martin FJ, Amode MR, Aneja A. et al. Ensembl 2023. *Nucleic Acids Res* 2023;51:D933–41. 10.1093/nar/gkac958. 36318249 PMC9825606

[77] Harrow J, Frankish A, Gonzalez JM. et al. GENCODE: the reference human genome annotation for the ENCODE project. *Genome Res* 2012;22:1760–74. 10.1101/gr.135350.111. 22955987 PMC3431492

[78] Leebens-Mack JH, Barker MS, Carpenter EJ. et al. One thousand plant transcriptomes and the phylogenomics of green plants. *Nature* 2019;574:679–85. 31645766 10.1038/s41586-019-1693-2 PMC6872490

[79] Haeussler M, Zweig AS, Tyner C. et al. The UCSC genome browser database: 2019 update. *Nucleic Acids Res* 2019;47:D853–8. 10.1093/nar/gky1095. 30407534 PMC6323953

[80] Chu Y, Yu D, Li Y. et al. A 5′ UTR language model for decoding untranslated regions of mRNA and function predictions. *Nat Mach Intell* 2024;6:449–60. 10.1038/s42256-024-00823-9. 38855263 PMC11155392

[81] Sample PJ, Wang B, Reid DW. et al. Human 5’ UTR design and variant effect prediction from a massively parallel translation assay. *Nat Biotechnol* 2019;37:803–9. 10.1038/s41587-019-0164-5. 31267113 PMC7100133

[82] Cao J, Novoa EM, Zhang Z. et al. High-throughput 5’ UTR engineering for enhanced protein production in non-viral gene therapies. *Nat Commun* 2021;12:4138. 10.1038/s41467-021-24436-7. 34230498 PMC8260622

[83] Cavallo L, Kleinjung J, Fraternali F. POPS: a fast algorithm for solvent accessible surface areas at atomic and residue level. *Nucleic Acids Res* 2003;31:3364–6. 12824328 10.1093/nar/gkg601 PMC169007

[84] Tan Z, Fu Y, Sharma G. et al. TurboFold II: RNA structural alignment and secondary structure prediction informed by multiple homologs. *Nucleic Acids Res* 2017;45:11570–81. 10.1093/nar/gkx815. 29036420 PMC5714223

[85] Sloma MF, Mathews DH. Exact calculation of loop formation probability identifies folding motifs in RNA secondary structures. *RNA* 2016;22:1808–18. 27852924 10.1261/rna.053694.115 PMC5113201

[86] Danaee P, Rouches M, Wiley M. et al. bpRNA: large-scale automated annotation and analysis of RNA secondary structure. *Nucleic Acids Res* 2018;46:5381–94. 10.1093/nar/gky285. 29746666 PMC6009582

[87] Singh J, Hanson J, Paliwal K. et al. RNA secondary structure prediction using an ensemble of two-dimensional deep neural networks and transfer learning. *Nat Commun* 2019;10:5407. 10.1038/s41467-019-13395-9. 31776342 PMC6881452

[88] Singh J, Paliwal K, Zhang T. et al. Improved RNA secondary structure and tertiary base-pairing prediction using evolutionary profile, mutational coupling and two-dimensional transfer learning. *Bioinformatics* 2021;37:2589–600. 10.1093/bioinformatics/btab165. 33704363

[89] Leontis NB, Zirbel CL. Nonredundant 3D structure datasets for RNA knowledge extraction and benchmarking. In: Leontis N, Westhof E, (eds.), *RNA 3D Structure Analysis and Prediction*. Berlin Heidelberg: Springer, 2012, 281–98.

[90] Singh J, Paliwal K, Singh J. et al. RNA backbone torsion and Pseudotorsion angle prediction using dilated convolutional neural networks. *J Chem Inf Model* 2021;61:2610–22. 10.1021/acs.jcim.1c00153. 34037398

[91] Wilm A, Mainz I, Steger G. An enhanced RNA alignment benchmark for sequence alignment programs. *Algorithms Mol Biol* 2006;1:19. 10.1186/1748-7188-1-19. 17062125 PMC1635699

[92] Mathews DH . How to benchmark RNA secondary structure prediction accuracy. *Methods* 2019;162-163:60–7. 10.1016/j.ymeth.2019.04.003. 30951834 PMC7202366

[93] Scalzitti N, Kress A, Orhand R. et al. Spliceator: multi-species splice site prediction using convolutional neural networks. *BMC Bioinform* 2021;22:561. 10.1186/s12859-021-04471-3. PMC8609763 34814826

[94] Song Z, Huang D, Song B. et al. Attention-based multi-label neural networks for integrated prediction and interpretation of twelve widely occurring RNA modifications. *Nat Commun* 2021;12:4011. 10.1038/s41467-021-24313-3. 34188054 PMC8242015

[95] Tang Y, Chen K, Song B. et al. m6A-atlas: a comprehensive knowledgebase for unraveling the N6-methyladenosine (m6A) epitranscriptome. *Nucleic Acids Res* 2020;49:D134–43. 10.1093/nar/gkaa692. PMC7779050 32821938

[96] Han Y, Zhang S-W. ncRPI-LGAT: prediction of ncRNA-protein interactions with line graph attention network framework. *Comput Struct Biotechnol J* 2023;21:2286–95. 37035546 10.1016/j.csbj.2023.03.027 PMC10073990

[97] Wen M, Cong P, Zhang Z. et al. DeepMirTar: a deep-learning approach for predicting human miRNA targets. *Bioinformatics* 2018;34:3781–7. 10.1093/bioinformatics/bty424. 29868708

[98] Kang Q, Meng J, Cui J. et al. PmliPred: a method based on hybrid model and fuzzy decision for plant miRNA–lncRNA interaction prediction. *Bioinformatics* 2020;36:2986–92. 10.1093/bioinformatics/btaa074. 32087005

[99] Wang D, Zhang Z, Jiang Y. et al. DM3Loc: multi-label mRNA subcellular localization prediction and analysis based on multi-head self-attention mechanism. *Nucleic Acids Res* 2021;49:e46–6. 33503258 10.1093/nar/gkab016 PMC8096227

[100] Wu L, Wang L, Hu S. et al. RNALocate v3.0: advancing the repository of RNA subcellular localization with dynamic analysis and prediction. *Nucleic Acids Res* 2024;53:D284–92. 10.1093/nar/gkae872. PMC11701552 39404071

[101] Goodstein DM, Shu S, Howson R. et al. Phytozome: a comparative platform for green plant genomics. *Nucleic Acids Res* 2012;40:D1178–86. 10.1093/nar/gkr944. 22110026 PMC3245001

[102] Kolekar P, Pataskar A, Kulkarni-Kale U. et al. IRESPred: web server for prediction of cellular and viral internal ribosome entry site (IRES). *Sci Rep* 2016;6:27436. 10.1038/srep27436. 27264539 PMC4893748

[103] Weingarten-Gabbay S, Elias-Kirma S, Nir R. et al. Systematic discovery of cap-independent translation sequences in human and viral genomes. *Science* 2016;351:aad4939. 10.1126/science.aad4939. 26816383

[104] Zhao J, Li Y, Wang C. et al. IRESbase: a comprehensive database of experimentally validated internal ribosome entry sites. *Genomics Proteomics Bioinformatics* 2020;18:129–39. 10.1016/j.gpb.2020.03.001. 32512182 PMC7646085

[105] Mokrejš M, Masek T, Vopálensky V. et al. IRESite—A tool for the examination of viral and cellular internal ribosome entry sites. *Nucleic Acids Res* 2010;38:D131–6. 10.1093/nar/gkp981. 19917642 PMC2808886

[106] Karollus A, Avsec Ž, Gagneur J. Predicting mean ribosome load for 5’UTR of any length using deep learning. *PLoS Comput Biol* 2021;17:e1008982. 10.1371/journal.pcbi.1008982. 33970899 PMC8136849

[107] Steinegger M, Söding J. MMseqs2 enables sensitive protein sequence searching for the analysis of massive data sets. *Nat Biotechnol* 2017;35:1026–8. 10.1038/nbt.3988. 29035372

[108] Fu L, Niu B, Zhu Z. et al. CD-HIT: accelerated for clustering the next-generation sequencing data. *Bioinformatics* 2012;28:3150–2. 10.1093/bioinformatics/bts565. 23060610 PMC3516142

[109] Nawrocki EP, Eddy SR. Infernal 1.1: 100-fold faster RNA homology searches. *Bioinformatics* 2013;29:2933–5. 10.1093/bioinformatics/btt509. 24008419 PMC3810854

[110] Lorenz R, Bernhart SH, Höner zu Siederdissen C. et al. ViennaRNA package 2.0. *Algorithms Mol Biol* 2011;6:26. 10.1186/1748-7188-6-26. 22115189 PMC3319429

[111] Rose PW, Prlić A, Altunkaya A. et al. The RCSB protein data bank: integrative view of protein, gene and 3D structural information. *Nucleic Acids Res* 2017;45:D271–81. 10.1093/nar/gkw1000. 27794042 PMC5210513

[112] Menzel P, Gorodkin J, Stadler PF. The tedious task of finding homologous noncoding RNA genes. *RNA* 2009;15:2075–82. 10.1261/rna.1556009. 19861422 PMC2779685

