# Supplementary material for: Zero-shot benchmarking of RNA language models in structural, functional, and evolutionary learning
Source: Brief Bioinform. 2026 Mar 6;27(2):bbag098. doi: 10.1093/bib/bbag098 (PMC12963973; doi:10.1093/bib/bbag098)
Supplement: bbag098_Supplymentary_Information_20260209 [file bbag098_supplymentary_information_20260209.docx]

**Zero-shot Benchmarking of RNA Language Models in Structural, Functional, and Evolutionary Learning**

He Wang^1^, Yikun Zhang^1,2^, Jie Chen^2^, Jian Zhan^1,3,4^**^*^**, Yaoqi Zhou^1^**^*^**

^1^ Institute of Systems and Physical Biology, Shenzhen Bay Laboratory, Shenzhen 518107, China

^2^ School of Electronic and Computer Engineering, Peking University, Shenzhen 518055, China

^3^ Ribopeutic (Shenzhen) Co., Ltd., Futian, Shenzhen, Guangdong Province, 518000, China

^4^ Ribopeutic Inc., Qiantang, Hangzhou, Zhejiang Province, 310018, China

*** To whom correspondence should be addressed. Tel: +86 755 26849275; Email:** [**zhouyq@szbl.ac.cn**](mailto:zhouyq@szbl.ac.cn)

**Correspondence may also be addressed to Jian Zhan. Tel: +86 755 26849280; Email: zhanjian@szbl.ac.cn**

**Additional Information on BERT-Based RNA Language Models**

As shown in **Figure 1** in the manuscript, the basic network for BERT-based RNA language models consisting of three main steps: the input layer (including masking and embedding), the encoding layer, and the output processing^1^. First, the input RNA sequence is segmented and converted into a series of tokens, e.g., four bases (“A”, “U”, “G”, and “C”). Next, during the masking process, typically 15% of the tokens from each training sequence were randomly selected. Of these, 80% are masked, i.e., replaced with “[mask]” tokens, 10% are replaced with randomly selected tokens from the vocabulary, and the remaining 10% remain unchanged. Then, these tokens are converted into word vectors and fed into the encoding layer, which is the core part of the BERT model and consists of stacked multiple Transformer encoders. The whole encoding process can be viewed as a special aggregation operation on the input sequence to obtain a more comprehensive and richer representation. Finally, the output processing is determined by the masked language model (MLM)^1^, which requires the BERT to predict the original form of those masked tokens, prompting the model to learn the ability to make predictions with incomplete contextual information. The training process of the RNA LMs usually consists of two stages: pre-training and fine-tuning. The pre-training stage typically requires large-scale RNA sequences to ensure that the model learns the general semantic representation. Attention maps and output sequence embeddings of the pretrained model can be extracted for downstream RNA structure and function prediction. In the fine-tuning stage, the foundation model can be further fine-tuned according to specific datasets to obtain better performance than original model.

**Additional Information in Methods**

**Calculation of the cosine similarity between sequence embeddings**

For each RNA sequence, embeddings were first extracted from the corresponding language model. To obtain a fixed-length representation that is independent of sequence length, the raw embeddings were transformed using a Fast Fourier Transform (FFT)–based procedure. Specifically, given an embedding matrix

$$x\in\mathbb{R}^{L\times D}$$

where $L$ denotes the sequence length and $D$ the embedding dimension, FFT was applied along the sequence-length dimension, followed by truncation to generate a representational vector

$$\mathbf{v}\in\mathbb{R}^{K},$$

where $K=128$ in this study.

For two sequences $A$and $B$, with corresponding representational vectors $\mathbf{v}_{A}$ and $\mathbf{v}_{B}$, the cosine similarity (CS) was computed as

$$\mathrm{CS}(A,B)=\frac{\mathbf{v}_{A}\cdot\mathbf{v}_{B}}{\parallel\mathbf{v}_{A}\parallel\text{ }\parallel\mathbf{v}_{B}\parallel}.$$

The cosine similarity ranges from −1 to 1, with higher values indicating greater similarity between the sequence representations.

For language models equipped with a dedicated classification token (CLS), we additionally evaluated a reference representation by directly using the final-layer CLS embedding as a sequence-level vector. Cosine similarity was computed analogously based on these CLS embeddings. This similarity measure was included as a reference.

**Calculation of the** **overlap ratio (OR)**

The overlap ratio (OR) was used to quantify the separability between two similarity distributions. OR is defined as the area of intersection between two probability density curves divided by the total area covered by their union under the same coordinate system. Its value ranges from 0 to 1, where OR = 0 indicates complete separation and OR = 1 indicates complete overlap between the two distributions.

In zero-shot comparison on RNA classification, OR was computed between the cosine similarity distributions of randomly sampled homologous sequence pairs within the same RNA family or with the same function and non-homologous sequence pairs between different RNA families or with different functions. For robustness, three independent random samplings were performed, and the final OR value was reported as the average across these replicates.

**Zero-shot fitness score calculation**

Zero-shot mutational fitness prediction was conducted following the evaluation protocol introduced in ProteinGym^2^ and subsequently adopted in RNAGym^3^. These approaches assume that the likelihood assigned by a language model reflects evolutionary and functional constraints encoded in natural sequences.

Let $x^{wt}=(x_{1},\ldots,x_{L})$ denote a wild-type sequence and let $x^{mut}$ denote a mutant sequence differing from $x^{wt}$at positions $M\subseteq\{1,\ldots,L\}$. For a language model parameterized by $\theta$, we denote by $p_{\theta}(x_{i}\mid x_{\backslash i})$, the conditional probability of nucleotide $x_{i}$ at position $i$, given the remaining sequence context ($x_{\backslash i}$). Following previous methods^3^, we consider two likelihood-based fitness scores, defined as follows.

(i) Wild-Type Conditioned Log-Likelihood ratio (WT-LLR)

The WT-LLR score is defined as^4^

$$Score=\sum_{i\in M} [\log p_{\theta}\left( x_{i}=x_{i}^{mut} | x^{wt} \right)-\log p_{\theta}\left( x_{i}=x_{i}^{wt} | x^{wt} \right)].$$

This score measures the relative preference of the language model for the mutant nucleotide compared to the wild-type base under an identical wild-type context. In practice, WT-LLR can be computed from a single forward pass of the wild-type sequence.

(ii) Pseudo-Log-Likelihood Difference (PLL-D)

The pseudo-log-likelihood of a sequence $x$ is defined as^5^

$$\log p_{\mathrm{PLL}}(x)=\sum_{i=1}^{L} \log p_{\theta}\left( x_{i} \mid x_{\setminus i} \right).$$

Based on this definition, the PLL-D fitness score is given by

$$Score=\sum_{i=1}^{L} \log p_{\theta}\left( x_{i}=x_{i}^{mut} | x_{\backslash i}^{mut} \right)-\sum_{i=1}^{L} \log p_{\theta}\left( x_{i}=x_{i}^{wt} | x_{\backslash i}^{wt} \right)$$

This score represents the difference in pseudo-log-likelihoods between the mutant and wild-type sequences and captures the global effect of mutations across the entire sequence.

**Supplementary Table 1.** Download locations and version numbers for all baseline RNA and DNA language models evaluated in this study.

| **Model** | **Download URL** | **Download Date** | **Version** |
| --- | --- | --- | --- |
| **RNA LMs** |  |  |  |
| 3UTRBERT | https://github.com/yangyn533/3UTRBERT | 2025/12/13 | 3-new-12w-0 (**K3**)/4-new-12w-0 (**K4**)/5-new-12w-0 (**K5**)/6-new-12w-0 (**K6**) |
| AIDO.RNA | https://github.com/genbio-ai/AIDO | 2024/12/11 | AIDO.RNA-650M (**650M**)/AIDO.RNA-1B600M (**1.6B**) |
| BiRNA-BERT | https://huggingface.co/buetnlpbio/birna-bert | 2024/10/8 | - |
| DGRNA | https://github.com/TimothyChen225/DGRNA | 2025/12/17 | - |
| ERNIE-RNA | https://github.com/Bruce-ywj/ERNIE-RNA | 2024/7/26 | - |
| GenerRNA | https://huggingface.co/pfnet/GenerRNA | 2025/12/13 | - |
| LAMAR | https://huggingface.co/zhw-e8/LAMAR/tree/main | 2025/6/23 | mammalian80D_2048len1mer1sw_80M (**2k**)/mammalian80D_4096len1mer1sw_80M (**4k**) |
| LucaOne | https://huggingface.co/LucaGroup | 2025/12/12 | LucaOne-default-step17.6M (**D17.6**)/LucaOne-default-step36M (**D36.0**)/LucaOne-gene-step36.8M (**G36.8**) |
| MP-RNA | https://huggingface.co/yangheng/MP-RNA | 2024/11/27 | - |
| PlantRNA-FM | https://huggingface.co/yangheng/PlantRNA-FM | 2025/12/5 | - |
| ProtRNA | https://github.com/roxie-zhang/ProtRNA | 2024/9/24 | - |
| RFamLlama | https://huggingface.co/jinyuan22 | 2025/12/13 | RFamLlama-large (**Large**)/ RFamLlama-base (**Base**) |
| RiNALMo | https://github.com/lbcb-sci/RiNALMo | 2024/7/26 | micro (**micro**)/mega (**mega**)/giga (**giga**) |
| RNABERT | https://huggingface.co/multimolecule/rnabert | 2024/11/27 | - |
| RNAErnie | https://github.com/CatIIIIIIII/RNAErnie | 2024/7/26 | - |
| RNA-FM | https://github.com/ml4bio/RNA-FM | 2024/7/26 | - |
| RNA-km | https://github.com/gongtiansu/RNA-km | 2024/9/6 | - |
| RNA-MSM | https://github.com/yikunpku/RNA-MSM | 2024/10/8 | - |
| SpliceBERT | https://github.com/chenkenbio/SpliceBERT | 2024/8/11 | SpliceBERT.1024nt |
| Uni-RNA | https://github.com/ComDec/unirna_tf | 2025/12/17 | unirna_L12 (**L12**)/unirna_L16 (**L16**)/unirna_L8 (**L8**) |
| UTR_LM | https://github.com/a96123155/UTR-LM | 2024/8/12 | - |
| **DNA LMs** |  |  |  |
| DNABERT | https://github.com/jerryji1993/DNABERT | 2024/9/27 | 3-new-12w-0 (**k=3**)/6-new-12w-0 (**k=6**) |
| DNABERT-2 | https://github.com/MAGICS-LAB/DNABERT_2 | 2024/8/13 | DNABERT-2-117M |
| EVO1.5 | https://huggingface.co/evo-design/evo-1.5-8k-base | 2025/12/17 | evo-1.5-8k-base (**8k_base**) |
| NT | https://github.com/instadeepai/nucleotide-transformer | 2024/8/26 | v2-250m-multi-species |
| EVO1 | https://huggingface.co/togethercomputer/evo-1-8k-base | 2025/12/17 | evo-1-8k-base (**8k_base**) |

Note: The abbreviations in parentheses in the “**Version**” column denote the version.

**Supplementary Table 2.** Composition of the ArchiveII dataset and its non-redundant subset.

| **RNA Type** | **Nums. of All** | **Nums. of Non-redundant** |
| --- | --- | --- |
| 5S rRNA | 1283 | 183 |
| 16S rRNA | 66 | 54 |
| 23S rRNA | 15 | 11 |
| tRNA | 557 | 319 |
| tmRNA | 462 | 197 |
| telomerase RNA | 35 | 17 |
| Group I intron | 74 | 56 |
| SRP RNA | 918 | 215 |
| RNase P RNA | 454 | 221 |
| ALL | 3864 | 1273 |

**Supplementary Table 3.** Availability of attention weights, embedding representations, and output logits for each language model.

| Model | Attetions \| SS | Embeddings \| Classification | Logits \| Fitness |
| --- | --- | --- | --- |
| **RNA LMs** |  |  |  |
| 3UTRBERT | ☑ | ☑ | ☑ |
| AIDO.RNA | ☑ | ☑ | ☑ |
| BiRNA-BERT | ☒ | ☑ | ☒ |
| DGRNA | ☒ | ☑ | ☑ |
| ERNIE-RNA | ☑ | ☑ | ☒ |
| GenerRNA | ☒ | ☑ | ☑ |
| LAMAR | ☑ | ☑ | ☑ |
| LucaOne | ☑ | ☑ | ☑ |
| MP-RNA | ☑ | ☑ | ☑ |
| PlantRNA-FM | ☑ | ☑ | ☑ |
| ProtRNA | ☑ | ☑ | ☑ |
| RFamLlama | ☑ | ☑ | ☑ |
| RiNALMo | ☑ | ☑ | ☑ |
| RNABERT | ☑ | ☑ | ☑ |
| RNAErnie | ☑ | ☑ | ☑ |
| RNA-FM | ☑ | ☑ | ☑ |
| RNA-km | ☑ | ☑ | ☒ |
| RNA-MSM | ☑ | ☑ | ☑ |
| SpliceBERT | ☑ | ☑ | ☑ |
| Uni-RNA | ☑ | ☑ | ☑ |
| UTR_LM | ☑ | ☑ | ☑ |
| **DNA LMs** |  |  |  |
| DNABERT | ☑ | ☑ | ☑ |
| DNABERT-2 | ☒ | ☑ | ☑ |
| EVO1.5 | ☒ | ☒ | ☑ |
| NT | ☒ | ☑ | ☑ |
| EVO1 | ☒ | ☒ | ☑ |

Note: Extracted attention weights, embedding representations, and output logits were used for zero-shot secondary structure prediction, zero-shot RNA classification, and zero-shot fitness prediction, respectively. Although embeddings and logits are available for RNA-MSM, related downstream analyses were not performed due to the huge computational cost for homology search.

**Supplementary Table 4**. Zero-shot RNA secondary structure prediction performance on the TS dataset (70 PDB structures), measured by median F1 score and median Matthews correlation coefficient (MCC), and ranked by median MCC (ascending order, ↑).

| **Model** | **F1 score** | | **MCC** | |
| --- | --- | --- | --- | --- |
|  | **Median** | **Mean ± Std** | **Median (↑)** | **Mean ± Std** |
| **DNA LMs** |  |  |  |  |
| DNABERT (k=3) | 0.000 | 0.011±0.019 | -0.006 | -0.008±0.025 |
| DNABERT (k=6) | **0.010** | **0.017±0.021** | **0.003** | **0.001±0.024** |
| **Class III RNA LMs** |  |  |  |  |
| LucaOne (D36.0M) | 0.141 | 0.211±0.200 | 0.135 | 0.214±0.203 |
| LucaOne (D17.6M) | 0.175 | 0.224±0.194 | 0.182 | 0.228±0.196 |
| LucaOne (G36.8M) | **0.197** | **0.253±0.232** | **0.228** | **0.268±0.238** |
| **Class II RNA LMs** |  |  |  |  |
| 3UTRBERT (K4) | 0.000 | 0.018±0.027 | -0.005 | 0.001±0.026 |
| 3UTRBERT (K5) | 0.003 | 0.023±0.034 | -0.002 | 0.007±0.040 |
| 3UTRBERT (K3) | 0.013 | 0.022±0.027 | -0.002 | 0.004±0.030 |
| UTR_LM | 0.022 | 0.029±0.032 | 0.006 | 0.016±0.037 |
| 3UTRBERT (K6) | 0.021 | 0.022±0.012 | 0.008 | 0.003±0.032 |
| SpliceBERT | 0.034 | 0.043±0.039 | 0.022 | 0.033±0.045 |
| PlantRNA-FM | 0.124 | 0.160±0.137 | 0.116 | 0.155±0.137 |
| MP-RNA | **0.507** | **0.507±0.201** | **0.515** | **0.510±0.200** |
| **Class I RNA LMs** |  |  |  |  |
| *RNABERT | 0.000 | 0.022±0.032 | -0.001 | 0.010±0.029 |
| LAMAR (4k) | 0.025 | 0.033±0.040 | 0.000 | 0.025±0.039 |
| RFamLlama (Base) | 0.034 | 0.042±0.040 | 0.029 | 0.031±0.045 |
| LAMAR (2k) | 0.037 | 0.037±0.021 | 0.031 | 0.029±0.027 |
| RFamLlama (Large) | 0.040 | 0.052±0.057 | 0.036 | 0.042±0.058 |
| RNA-FM | 0.180 | 0.267±0.239 | 0.186 | 0.273±0.239 |
| Uni-RNA (L8) | 0.225 | 0.257±0.197 | 0.219 | 0.258±0.202 |
| RNAErnie | 0.238 | 0.288±0.223 | 0.236 | 0.290±0.221 |
| Uni-RNA (L12) | 0.247 | 0.258±0.198 | 0.244 | 0.258±0.203 |
| RiNALMo (micro) | 0.256 | 0.268±0.184 | 0.268 | 0.275±0.184 |
| Uni-RNA (L16) | 0.307 | 0.307±0.197 | 0.308 | 0.311±0.200 |
| RiNALMo (mega) | 0.315 | 0.342±0.225 | 0.317 | 0.342±0.225 |
| ProtRNA | 0.298 | 0.333±0.236 | 0.323 | 0.351±0.237 |
| ERNIE-RNA | 0.259 | 0.300±0.234 | 0.356 | 0.351±0.230 |
| RiNALMo (giga) | 0.404 | 0.414±0.255 | 0.416 | 0.423±0.257 |
| AIDO.RNA (650M) | 0.438 | 0.402±0.234 | 0.469 | 0.427±0.235 |
| RNA-km | 0.473 | 0.492±0.236 | 0.489 | 0.501±0.233 |
| AIDO.RNA (1.6B) | 0.579 | 0.508±0.263 | 0.595 | 0.518±0.258 |
| RNA-MSM | **0.631** | **0.582±0.217** | **0.634** | **0.588±0.217** |

Note: *RNABERT was evaluated on a reduced number of PDB structures (69 PDBs in TS) due to sequence length constraints (≤ 440 nt). Bold font indicates the best-performing model within each language model category. Model variants are specified in parentheses.

**Supplementary Table 5**. Zero-shot RNA secondary structure prediction performance on the TS-Hard dataset (15 PDB structures), measured by median F1 score and median Matthews correlation coefficient (MCC), and ranked by median MCC (ascending order, ↑).

| **Model** | **F1 score** | | **MCC** | |
| --- | --- | --- | --- | --- |
|  | **Median** | **Mean ± Std** | **Median (↑)** | **Mean ± Std** |
| **DNA LMs** |  |  |  |  |
| DNABERT (k=3) | 0.000 | 0.003±0.007 | -0.005 | -0.010±0.010 |
| DNABERT (k=6) | **0.011** | **0.011±0.011** | **0.003** | **-0.002±0.018** |
| **Class III RNA LMs** |  |  |  |  |
| LucaOne (D36.0M) | 0.104 | 0.187±0.162 | 0.110 | 0.196±0.173 |
| LucaOne (D17.6M) | 0.130 | 0.185±0.157 | 0.127 | 0.189±0.159 |
| LucaOne (G36.8M) | **0.182** | **0.246±0.227** | **0.185** | **0.261±0.230** |
| **Class II RNA LMs** |  |  |  |  |
| 3UTRBERT (K4) | 0.000 | 0.015±0.025 | -0.005 | 0.003±0.026 |
| 3UTRBERT (K3) | 0.008 | 0.014±0.015 | -0.001 | 0.000±0.014 |
| 3UTRBERT (K5) | 0.000 | 0.014±0.019 | -0.001 | 0.003±0.019 |
| UTR_LM | 0.011 | 0.016±0.018 | 0.000 | 0.006±0.018 |
| 3UTRBERT (K6) | 0.017 | 0.019±0.010 | 0.009 | 0.007±0.025 |
| SpliceBERT | 0.034 | 0.030±0.023 | 0.025 | 0.020±0.026 |
| PlantRNA-FM | 0.085 | 0.109±0.096 | 0.082 | 0.104±0.094 |
| MP-RNA | **0.519** | **0.545±0.217** | **0.516** | **0.547±0.215** |
| **Class I RNA LMs** |  |  |  |  |
| *RNABERT | 0.000 | 0.014±0.026 | -0.001 | 0.007±0.023 |
| LAMAR (4k) | 0.000 | 0.020±0.035 | 0.000 | 0.014±0.029 |
| RFamLlama (Base) | 0.020 | 0.019±0.015 | 0.015 | 0.009±0.021 |
| LAMAR (2k) | 0.035 | 0.035±0.020 | 0.033 | 0.032±0.025 |
| RFamLlama (Large) | 0.047 | 0.047±0.040 | 0.042 | 0.039±0.041 |
| RNA-FM | 0.148 | 0.224±0.228 | 0.164 | 0.231±0.228 |
| RiNALMo (micro) | 0.180 | 0.190±0.121 | 0.188 | 0.204±0.135 |
| RiNALMo (mega) | 0.197 | 0.265±0.204 | 0.198 | 0.265±0.210 |
| Uni-RNA (L12) | 0.203 | 0.225±0.152 | 0.199 | 0.221±0.153 |
| Uni-RNA (L8) | 0.215 | 0.262±0.143 | 0.217 | 0.264±0.148 |
| RNAErnie | 0.231 | 0.240±0.152 | 0.237 | 0.243±0.152 |
| ProtRNA | 0.276 | 0.280±0.189 | 0.295 | 0.301±0.195 |
| Uni-RNA (L16) | 0.286 | 0.284±0.163 | 0.323 | 0.286±0.162 |
| RiNALMo (giga) | 0.337 | 0.365±0.214 | 0.347 | 0.375±0.222 |
| ERNIE-RNA | 0.267 | 0.291±0.185 | 0.369 | 0.341±0.176 |
| RNA-km | 0.404 | 0.444±0.206 | 0.410 | 0.453±0.201 |
| AIDO.RNA (650M) | 0.331 | 0.331±0.217 | 0.412 | 0.365±0.227 |
| AIDO.RNA (1.6B) | 0.600 | 0.512±0.262 | 0.618 | 0.520±0.263 |
| RNA-MSM | **0.679** | **0.645±0.136** | **0.690** | **0.654±0.138** |

Note: *RNABERT was evaluated on a reduced number of PDB structures (14 PDBs in TS) due to sequence length constraints (≤ 440 nt). Bold font indicates the best-performing model within each language model category. Model variants are specified in parentheses.

**Supplementary Table 6**. Zero-shot RNA family classification on the RfamSample dataset using FFT-based embeddings, measured by mean F1-score, Matthews correlation coefficient (MCC), area under the receiver operating characteristic curve (AUC) and the overlap ratio (OR), and ranked by mean 1 − OR (ascending order, ↑).

| **Model** | **F1** | **MCC** | **AUC** | **1 − OR (↑)** |
| --- | --- | --- | --- | --- |
| **DNA LMs** |  |  |  |  |
| *DNABERT (k=6) | 0.661±0.001 | 0.085±0.002 | 0.511±0.001 | 0.324±0.004 |
| DNABERT-2 | 0.671±0.000 | 0.132±0.011 | 0.655±0.001 | 0.404±0.003 |
| NT | 0.709±0.001 | 0.352±0.001 | 0.772±0.001 | 0.575±0.001 |
| *DNABERT (k=3) | **0.721±0.001** | **0.373±0.010** | **0.779±0.002** | **0.585±0.003** |
| **Class III RNA LMs** |  |  |  |  |
| LucaOne (G36.8M) | 0.685±0.001 | 0.219±0.016 | 0.652±0.002 | 0.418±0.004 |
| LucaOne (D36.0M) | 0.713±0.000 | 0.359±0.014 | 0.772±0.001 | 0.583±0.002 |
| LucaOne (D17.6M) | **0.760±0.001** | **0.493±0.006** | **0.828±0.001** | **0.677±0.001** |
| **Class II RNA LMs** |  |  |  |  |
| *3UTRBERT (K6) | 0.670±0.001 | 0.134±0.001 | 0.670±0.001 | 0.433±0.002 |
| *3UTRBERT (K4) | 0.676±0.001 | 0.176±0.009 | 0.691±0.001 | 0.486±0.000 |
| *3UTRBERT (K3) | 0.696±0.001 | 0.306±0.008 | 0.740±0.001 | 0.534±0.001 |
| *3UTRBERT (K5) | 0.687±0.002 | 0.253±0.012 | 0.521±0.001 | 0.545±0.002 |
| SpliceBERT | 0.706±0.001 | 0.341±0.001 | 0.762±0.001 | 0.567±0.002 |
| PlantRNA-FM | 0.715±0.001 | 0.369±0.002 | 0.779±0.002 | 0.587±0.003 |
| MP-RNA | **0.731±0.001** | **0.420±0.001** | 0.792±0.000 | 0.619±0.000 |
| UTR_LM | 0.729±0.000 | 0.405±0.001 | **0.801±0.000** | **0.631±0.000** |
| **Class I RNA LMs** |  |  |  |  |
| RFamLlama (Base) | 0.667±0.000 | 0.000±0.000 | 0.660±0.001 | 0.000±0.000 |
| RFamLlama (Large) | 0.744±0.001 | 0.514±0.002 | 0.685±0.001 | 0.000±0.000 |
| GenerRNA | 0.662±0.000 | 0.044±0.000 | 0.561±0.002 | 0.292±0.004 |
| DGRNA | 0.719±0.001 | 0.394±0.002 | 0.781±0.001 | 0.600±0.002 |
| BiRNA-BERT (BPE) | 0.739±0.000 | 0.425±0.014 | 0.806±0.001 | 0.619±0.002 |
| RNA-km | 0.756±0.001 | 0.486±0.002 | 0.830±0.001 | 0.678±0.002 |
| Uni-RNA (L16) | 0.759±0.001 | 0.480±0.003 | 0.828±0.001 | 0.683±0.002 |
| LAMAR (2k) | 0.769±0.001 | 0.510±0.002 | 0.842±0.001 | 0.691±0.002 |
| BiRNA-BERT (NUC) | 0.775±0.000 | 0.525±0.006 | 0.854±0.000 | 0.703±0.002 |
| Uni-RNA (L8) | 0.778±0.001 | 0.529±0.001 | 0.852±0.001 | 0.707±0.001 |
| RiNALMo (micro) | 0.774±0.001 | 0.524±0.003 | 0.819±0.001 | 0.708±0.002 |
| *RNAErnie | 0.775±0.001 | 0.532±0.001 | 0.854±0.001 | 0.709±0.000 |
| LAMAR (4k) | 0.783±0.000 | 0.546±0.001 | 0.862±0.001 | 0.717±0.001 |
| Uni-RNA (L12) | 0.779±0.001 | 0.535±0.001 | 0.849±0.000 | 0.723±0.001 |
| *RNABERT | 0.794±0.001 | 0.586±0.001 | 0.777±0.002 | 0.724±0.001 |
| RiNALMo (mega) | 0.785±0.001 | 0.531±0.002 | 0.758±0.001 | 0.744±0.001 |
| RiNALMo (giga) | 0.824±0.001 | 0.657±0.001 | 0.896±0.001 | 0.790±0.001 |
| AIDO.RNA (1.6B) | 0.859±0.001 | 0.717±0.001 | 0.931±0.001 | 0.836±0.001 |
| AIDO.RNA (650M) | 0.873±0.002 | 0.745±0.002 | 0.944±0.001 | 0.854±0.002 |
| ERNIE-RNA | 0.856±0.001 | 0.746±0.001 | 0.909±0.001 | 0.858±0.001 |
| ProtRNA | 0.881±0.001 | 0.761±0.001 | **0.953±0.000** | 0.862±0.001 |
| RNA-FM | **0.889±0.000** | **0.771±0.001** | **0.953±0.001** | **0.878±0.001** |

Note: “*” denotes that sequences exceeding 440/512 nt were excluded from evaluation due to the requirement of the model.

**Supplementary Table 7.** Zero-shot RNA type classification on the ArchiveII-Nr dataset using FFT-based embeddings, measured by mean F1 score, Matthews correlation coefficient (MCC), area under the receiver operating characteristic curve (AUC) and the overlap ratio (OR), and ranked by mean 1 − OR (ascending order, ↑).

| **Model** | **F1** | **MCC** | **AUC** | **1 − OR (↑)** |
| --- | --- | --- | --- | --- |
| **DNA LMs** |  |  |  |  |
| DNABERT (k=6) | 0.654±0.001 | 0.027±0.001 | 0.503±0.001 | 0.237±0.002 |
| NT | **0.672±0.001** | **0.113±0.003** | **0.656±0.001** | 0.346±0.002 |
| DNABERT (k=3) | 0.667±0.000 | -0.002±0.001 | 0.637±0.001 | 0.352±0.002 |
| DNABERT-2 | 0.668±0.000 | 0.078±0.018 | 0.643±0.001 | **0.369±0.002** |
| **Class III RNA LMs** |  |  |  |  |
| LucaOne (G36.8M) | 0.666±0.001 | 0.090±0.001 | 0.574±0.001 | 0.367±0.001 |
| LucaOne (D36.0M) | 0.667±0.000 | 0.069±0.001 | 0.601±0.001 | 0.398±0.004 |
| LucaOne (D17.6M) | **0.677±0.000** | **0.244±0.001** | **0.633±0.002** | **0.511±0.002** |
| **Class II RNA LMs** |  |  |  |  |
| 3UTRBERT (K5) | 0.657±0.000 | 0.073±0.001 | 0.469±0.001 | 0.243±0.002 |
| 3UTRBERT (K6) | 0.667±0.000 | 0.012±0.001 | 0.596±0.001 | 0.266±0.003 |
| 3UTRBERT (K3) | 0.667±0.000 | 0.015±0.003 | 0.623±0.002 | 0.307±0.005 |
| UTR_LM | 0.669±0.000 | 0.077±0.001 | 0.650±0.001 | 0.354±0.003 |
| 3UTRBERT (K4) | 0.667±0.000 | 0.032±0.001 | 0.647±0.001 | 0.371±0.002 |
| PlantRNA-FM | 0.667±0.000 | 0.016±0.007 | 0.673±0.001 | 0.416±0.002 |
| SpliceBERT | 0.712±0.000 | 0.312±0.001 | 0.731±0.002 | 0.487±0.003 |
| MP-RNA | **0.719±0.001** | **0.336±0.002** | **0.761±0.001** | **0.539±0.001** |
| **Class I RNA LMs** |  |  |  |  |
| RFamLlama (Base) | 0.667±0.000 | 0.000±0.000 | 0.613±0.001 | 0.000±0.000 |
| RFamLlama (Large) | 0.667±0.000 | 0.000±0.000 | 0.654±0.002 | 0.000±0.000 |
| GenerRNA | 0.657±0.001 | -0.003±0.002 | 0.489±0.001 | 0.146±0.006 |
| RiNALMo (mega) | 0.672±0.001 | 0.198±0.001 | 0.672±0.000 | 0.472±0.001 |
| RNA-km | 0.701±0.001 | 0.303±0.018 | 0.642±0.001 | 0.480±0.001 |
| BiRNA-BERT (BPE) | 0.685±0.000 | 0.238±0.001 | 0.724±0.001 | 0.493±0.001 |
| LAMAR (4k) | 0.715±0.001 | 0.334±0.001 | 0.743±0.002 | 0.522±0.002 |
| LAMAR (2k) | 0.724±0.001 | 0.358±0.005 | 0.754±0.000 | 0.542±0.000 |
| RiNALMo (giga) | 0.667±0.000 | 0.000±0.000 | 0.705±0.001 | 0.553±0.001 |
| RNAErnie | 0.713±0.001 | 0.367±0.011 | 0.765±0.001 | 0.572±0.002 |
| DGRNA | 0.698±0.001 | 0.369±0.002 | 0.753±0.001 | 0.580±0.001 |
| *RNABERT | 0.719±0.001 | 0.363±0.001 | 0.732±0.002 | 0.585±0.002 |
| Uni-RNA (L8) | 0.751±0.001 | 0.444±0.005 | 0.779±0.001 | 0.607±0.001 |
| Uni-RNA (L12) | 0.747±0.001 | 0.428±0.003 | 0.799±0.002 | 0.614±0.003 |
| RiNALMo (micro) | 0.672±0.002 | 0.435±0.002 | 0.705±0.001 | 0.621±0.001 |
| Uni-RNA (L16) | 0.750±0.001 | 0.440±0.002 | 0.798±0.001 | 0.623±0.002 |
| ERNIE-RNA | 0.721±0.002 | 0.404±0.004 | 0.774±0.002 | 0.628±0.003 |
| BiRNA-BERT (NUC) | 0.735±0.001 | 0.525±0.002 | 0.786±0.002 | 0.698±0.001 |
| AIDO.RNA (1.6B) | 0.767±0.001 | 0.532±0.001 | 0.805±0.001 | 0.718±0.001 |
| AIDO.RNA (650M) | 0.782±0.001 | 0.555±0.001 | 0.840±0.001 | 0.728±0.001 |
| RNA-FM | 0.789±0.001 | 0.568±0.002 | 0.872±0.001 | 0.750±0.001 |
| ProtRNA | **0.809±0.001** | **0.640±0.002** | **0.888±0.001** | **0.780±0.002** |

Note: “*” denotes that sequences exceeding 440 nt were excluded from evaluation due to the requirement of the model.

**Supplementary Table 8**. Zero-shot RNA family classification on the RfamSample dataset using CLS-token representations, measured by mean F1 score, Matthews correlation coefficient (MCC), area under the receiver operating characteristic curve (AUC) and the overlap ratio (OR), and ranked by mean 1 − OR (ascending order, ↑).

| **Model** | **F1** | **MCC** | **AUC** | **1 − OR (↑)** |
| --- | --- | --- | --- | --- |
| **DNA LMs** |  |  |  |  |
| *DNABERT (k=6) | 0.668±0.001 | 0.096±0.002 | 0.664±0.001 | 0.390±0.001 |
| DNABERT-2 | 0.668±0.001 | 0.106±0.042 | 0.673±0.001 | 0.415±0.002 |
| *DNABERT (k=3) | 0.726±0.001 | 0.388±0.004 | 0.786±0.001 | 0.591±0.000 |
| NT | **0.733±0.001** | **0.422±0.002** | **0.806±0.001** | **0.629±0.000** |
| **Class III RNA LMs** |  |  |  |  |
| LucaOne (D36.0M) | 0.706±0.001 | 0.363±0.002 | 0.747±0.001 | 0.568±0.001 |
| LucaOne (G36.8M) | 0.728±0.001 | 0.377±0.002 | 0.788±0.001 | 0.635±0.002 |
| LucaOne (D17.6M) | **0.774±0.001** | **0.515±0.001** | **0.850±0.001** | **0.712±0.000** |
| **Class II RNA LMs** |  |  |  |  |
| *3UTRBERT (K6) | 0.692±0.001 | 0.277±0.002 | 0.740±0.001 | 0.528±0.002 |
| *3UTRBERT (K3) | 0.705±0.001 | 0.325±0.007 | 0.763±0.001 | 0.571±0.002 |
| PlantRNA-FM | 0.716±0.001 | 0.373±0.002 | 0.781±0.001 | 0.593±0.003 |
| *3UTRBERT (K4) | 0.722±0.001 | 0.392±0.008 | 0.795±0.001 | 0.626±0.001 |
| SpliceBERT | 0.735±0.001 | 0.426±0.002 | 0.804±0.001 | 0.633±0.002 |
| *3UTRBERT (K5) | 0.732±0.001 | 0.414±0.001 | 0.809±0.001 | 0.634±0.003 |
| UTR_LM | 0.739±0.001 | 0.445±0.002 | 0.813±0.001 | 0.649±0.001 |
| MP-RNA | **0.745±0.001** | **0.447±0.000** | **0.817±0.000** | **0.652±0.001** |
| **Class I RNA LMs** |  |  |  |  |
| RFamLlama (Base) | 0.667±0.000 | 0.000±0.000 | 0.654±0.001 | 0.000±0.000 |
| RFamLlama (Large) | 0.748±0.002 | 0.520±0.003 | 0.688±0.002 | 0.000±0.000 |
| GenerRNA | 0.667±0.000 | 0.004±0.001 | 0.627±0.002 | 0.313±0.003 |
| DGRNA | 0.732±0.001 | 0.418±0.002 | 0.800±0.001 | 0.630±0.001 |
| BiRNA-BERT (BPE) | 0.760±0.001 | 0.509±0.001 | 0.838±0.001 | 0.678±0.001 |
| Uni-RNA (L16) | 0.776±0.001 | 0.528±0.002 | 0.849±0.001 | 0.709±0.002 |
| Uni-RNA (L8) | 0.785±0.001 | 0.540±0.001 | 0.854±0.001 | 0.709±0.001 |
| LAMAR (2k) | 0.776±0.001 | 0.527±0.002 | 0.848±0.001 | 0.711±0.001 |
| LAMAR (4k) | 0.787±0.001 | 0.566±0.002 | 0.863±0.001 | 0.724±0.002 |
| RiNALMo (micro) | 0.741±0.001 | 0.407±0.002 | 0.815±0.001 | 0.727±0.002 |
| *RNABERT | 0.797±0.001 | 0.588±0.001 | 0.831±0.001 | 0.730±0.001 |
| BiRNA-BERT (NUC) | 0.794±0.001 | 0.569±0.001 | 0.873±0.000 | 0.734±0.001 |
| Uni-RNA (L12) | 0.789±0.002 | 0.582±0.003 | 0.862±0.001 | 0.738±0.002 |
| RNA-km | 0.804±0.001 | 0.594±0.004 | 0.886±0.001 | 0.750±0.001 |
| *RNAErnie | 0.801±0.001 | 0.594±0.002 | 0.880±0.000 | 0.751±0.001 |
| RiNALMo (mega) | 0.798±0.001 | 0.571±0.002 | 0.787±0.001 | 0.761±0.001 |
| RiNALMo (giga) | 0.836±0.000 | 0.667±0.001 | 0.901±0.000 | 0.803±0.001 |
| AIDO.RNA (1.6B) | 0.884±0.001 | 0.767±0.002 | 0.949±0.001 | 0.868±0.001 |
| ERNIE-RNA | 0.886±0.001 | 0.779±0.001 | 0.907±0.001 | 0.873±0.001 |
| ProtRNA | 0.866±0.000 | 0.719±0.001 | 0.920±0.001 | 0.874±0.001 |
| AIDO.RNA (650M) | **0.901±0.001** | 0.802±0.002 | **0.964±0.00**1 | 0.889±0.001 |
| RNA-FM | 0.898±0.001 | **0.803±0.000** | 0.930±0.000 | **0.891±0.000** |

Note: “*” denotes that sequences exceeding 440/512 nt were excluded from evaluation due to the requirement of the model.

**Supplementary Table 9.** Zero-shot RNA type classification on the ArchiveII-Nr dataset using CLS-token representations, measured by mean F1 score, Matthews correlation coefficient (MCC), area under the receiver operating characteristic curve (AUC) and the overlap ratio (OR), and ranked by mean 1 − OR (ascending order, ↑).

| **Model** | **F1** | **MCC** | **AUC** | **1 − OR (↑)** |
| --- | --- | --- | --- | --- |
| **DNA LMs** |  |  |  |  |
| DNABERT (k=6) | 0.667±0.000 | 0.001±0.001 | 0.602±0.001 | 0.268±0.001 |
| DNABERT (k=3) | 0.667±0.000 | 0.000±0.000 | 0.626±0.001 | 0.329±0.003 |
| DNABERT-2 | 0.667±0.000 | 0.005±0.002 | 0.653±0.001 | 0.379±0.001 |
| NT | **0.682±0.000** | **0.214±0.001** | **0.686±0.001** | **0.413±0.002** |
| **Class III RNA LMs** |  |  |  |  |
| LucaOne (D36.0M) | 0.667±0.000 | 0.001±0.001 | 0.666±0.002 | 0.436±0.004 |
| LucaOne (D17.6M) | 0.668±0.000 | 0.076±0.001 | 0.711±0.001 | 0.542±0.002 |
| LucaOne (G36.8M) | **0.671±0.001** | **0.309±0.002** | **0.716±0.001** | **0.557±0.002** |
| **Class II RNA LMs** |  |  |  |  |
| 3UTRBERT (K3) | 0.667±0.000 | 0.025±0.003 | 0.621±0.001 | 0.291±0.003 |
| 3UTRBERT (K6) | 0.668±0.001 | 0.071±0.002 | 0.633±0.001 | 0.305±0.002 |
| 3UTRBERT (K4) | 0.667±0.000 | 0.003±0.003 | 0.640±0.001 | 0.326±0.003 |
| UTR_LM | 0.667±0.000 | 0.042±0.003 | 0.637±0.001 | 0.340±0.002 |
| 3UTRBERT (K5) | 0.667±0.000 | 0.007±0.004 | 0.638±0.000 | 0.353±0.002 |
| PlantRNA-FM | 0.667±0.000 | 0.023±0.031 | 0.673±0.001 | 0.398±0.001 |
| SpliceBERT | 0.709±0.001 | 0.308±0.002 | 0.727±0.001 | 0.469±0.002 |
| MP-RNA | **0.733±0.001** | **0.388±0.002** | **0.798±0.001** | **0.613±0.002** |
| **Class I RNA LMs** |  |  |  |  |
| RFamLlama (Base) | 0.667±0.000 | 0.000±0.000 | 0.613±0.002 | 0.000±0.000 |
| RFamLlama (Large) | 0.667±0.000 | 0.000±0.000 | 0.655±0.001 | 0.000±0.000 |
| GenerRNA | 0.667±0.000 | 0.001±0.002 | 0.527±0.001 | 0.134±0.003 |
| RiNALMo (mega) | 0.667±0.000 | 0.003±0.001 | 0.663±0.001 | 0.454±0.003 |
| LAMAR (4k) | 0.710±0.001 | 0.319±0.002 | 0.734±0.000 | 0.503±0.001 |
| LAMAR (2k) | 0.722±0.001 | 0.366±0.001 | 0.749±0.001 | 0.532±0.002 |
| BiRNA-BERT (BPE) | 0.702±0.001 | 0.317±0.001 | 0.756±0.000 | 0.555±0.002 |
| *RNABERT | 0.710±0.001 | 0.350±0.002 | 0.741±0.001 | 0.561±0.002 |
| RNA-km | 0.734±0.001 | 0.401±0.002 | 0.767±0.001 | 0.590±0.001 |
| RiNALMo (giga) | 0.675±0.001 | 0.332±0.001 | 0.721±0.001 | 0.591±0.002 |
| DGRNA | 0.710±0.001 | 0.422±0.001 | 0.771±0.001 | 0.609±0.001 |
| ERNIE-RNA | 0.714±0.001 | 0.352±0.003 | 0.770±0.002 | 0.611±0.002 |
| RNAErnie | 0.728±0.001 | 0.404±0.002 | 0.784±0.001 | 0.612±0.000 |
| Uni-RNA (L12) | 0.749±0.001 | 0.435±0.008 | 0.811±0.002 | 0.624±0.002 |
| Uni-RNA (L16) | 0.748±0.001 | 0.455±0.007 | 0.803±0.001 | 0.636±0.001 |
| Uni-RNA (L8) | 0.776±0.000 | 0.507±0.001 | 0.821±0.001 | 0.656±0.001 |
| RiNALMo (micro) | 0.684±0.002 | 0.401±0.002 | 0.716±0.001 | 0.659±0.001 |
| BiRNA-BERT (NUC) | 0.747±0.002 | 0.464±0.003 | 0.806±0.001 | 0.691±0.001 |
| AIDO.RNA (1.6B) | 0.802±0.001 | 0.591±0.002 | 0.891±0.001 | 0.756±0.001 |
| AIDO.RNA (650M) | 0.810±0.001 | 0.615±0.001 | 0.897±0.001 | 0.767±0.001 |
| RNA-FM | 0.803±0.001 | 0.635±0.001 | 0.861±0.001 | 0.771±0.001 |
| ProtRNA | **0.825±0.001** | **0.670±0.001** | **0.900±0.000** | **0.799±0.001** |

Note: “*” denotes that sequences exceeding 440 nt were excluded from evaluation due to the requirement of the model.

**Supplementary Table 10.** Zero-shot fitness prediction on 31 ncRNA assays from RNAGym using the WT-LLR score, measured by mean Matthews correlation coefficient (MCC), area under the receiver operating characteristic curve (AUC), and Spearman correlation coefficient (SR), and ranked by median SR (ascending order, ↑).

| **Model** | **MCC** | | **AUC** | | **SR** | |
| --- | --- | --- | --- | --- | --- | --- |
|  | **Median** | **Mean ± Std** | **Median** | **Mean ± Std** | **Median (↑)** | **Mean ± Std** |
| **Class III RNA LMs** |  |  |  |  |  |  |
| LucaOne (D17.6M) | **0.076** | 0.087±0.077 | 0.549 | 0.562±0.052 | 0.092 | 0.117±0.103 |
| LucaOne (G36.8M) | 0.059 | 0.089±0.077 | 0.548 | 0.563±0.053 | 0.104 | 0.125±0.102 |
| LucaOne (D36.0M) | 0.074 | **0.097±0.086** | **0.552** | **0.568±0.058** | **0.116** | **0.135±0.112** |
| **Class II RNA LMs** |  |  |  |  |  |  |
| PlantRNA-FM | 0.112 | 0.141±0.098 | 0.562 | 0.593±0.071 | 0.139 | 0.187±0.148 |
| SpliceBERT | **0.176** | **0.194±0.109** | **0.612** | **0.631±0.074** | 0.202 | **0.268±0.149** |
| MP-RNA | 0.143 | 0.165±0.105 | 0.609 | 0.617±0.076 | **0.215** | 0.240±0.159 |
| **Class I RNA LMs** |  |  |  |  |  |  |
| LAMAR (2k) | 0.062 | 0.072±0.059 | 0.529 | 0.547±0.039 | 0.063 | 0.093±0.078 |
| RNABERT | 0.044 | 0.071±0.067 | 0.532 | 0.549±0.046 | 0.075 | 0.100±0.086 |
| UTR_LM | 0.063 | 0.084±0.066 | 0.542 | 0.558±0.049 | 0.087 | 0.116±0.097 |
| RiNALMo (micro) | 0.092 | 0.137±0.112 | 0.553 | 0.586±0.076 | 0.087 | 0.175±0.158 |
| ProtRNA | 0.067 | 0.074±0.057 | 0.545 | 0.553±0.037 | 0.089 | 0.107±0.076 |
| RiNALMo (giga) | 0.095 | 0.137±0.118 | 0.547 | 0.587±0.079 | 0.095 | 0.175±0.165 |
| Uni-RNA (L12) | 0.108 | 0.138±0.117 | 0.553 | 0.582±0.081 | 0.096 | 0.170±0.165 |
| LAMAR (4k) | 0.070 | 0.104±0.086 | 0.546 | 0.570±0.059 | 0.100 | 0.139±0.115 |
| Uni-RNA (L16) | 0.100 | 0.140±0.113 | 0.554 | 0.584±0.080 | 0.111 | 0.173±0.164 |
| RiNALMo (mega) | 0.104 | 0.141±0.108 | 0.556 | 0.588±0.073 | 0.114 | 0.178±0.152 |
| RNAErnie | 0.099 | 0.125±0.105 | 0.568 | 0.581±0.071 | 0.133 | 0.162±0.148 |
| Uni-RNA (L8) | 0.115 | 0.149±0.102 | 0.573 | 0.591±0.076 | 0.135 | 0.186±0.156 |
| AIDO.RNA (650M) | 0.122 | 0.152±0.126 | 0.577 | 0.606±0.088 | 0.169 | 0.218±0.183 |
| DGRNA | 0.163 | **0.176±0.116** | **0.601** | **0.620±0.077** | 0.200 | **0.249±0.163** |
| RNA-FM | **0.172** | 0.168±0.125 | 0.600 | 0.609±0.081 | 0.207 | 0.226±0.170 |
| AIDO.RNA (1.6B) | 0.155 | 0.171±0.115 | 0.595 | 0.617±0.079 | **0.214** | 0.237±0.166 |

**Supplementary Table 11.** Zero-shot fitness prediction on 31 ncRNA assays from RNAGym using the PLL-D score, measured by mean Matthews correlation coefficient (MCC), area under the receiver operating characteristic curve (AUC), and Spearman correlation coefficient (SR), and ranked by median SR (ascendhing order, ↑).

| **Model** | **MCC** | | **AUC** | | **SR** | |
| --- | --- | --- | --- | --- | --- | --- |
|  | **Median** | **Mean ± Std** | **Median** | **Mean ± Std** | **Median (↑)** | **Mean ± Std** |
| **DNA LMs** |  |  |  |  |  |  |
| DNABERT (k=6) | 0.044 | 0.064±0.059 | 0.535 | 0.540±0.035 | 0.067 | 0.077±0.061 |
| DNABERT (k=3) | 0.048 | 0.054±0.038 | 0.531 | 0.535±0.025 | 0.068 | 0.073±0.051 |
| NT | 0.075 | 0.082±0.076 | 0.547 | 0.557±0.056 | 0.085 | 0.111±0.109 |
| DNABERT-2 | 0.061 | 0.069±0.052 | 0.542 | 0.543±0.031 | 0.086 | 0.085±0.056 |
| EVO1 (8k_base) | 0.064 | 0.081±0.063 | 0.538 | 0.556±0.048 | 0.105 | 0.119±0.100 |
| EVO1.5 (8k_base) | **0.130** | **0.132±0.088** | **0.586** | **0.588±0.056** | **0.178** | **0.179±0.114** |
| **Class III RNA LMs** |  |  |  |  |  |  |
| LucaOne (D36.0M) | 0.050 | 0.062±0.052 | 0.531 | 0.541±0.035 | 0.068 | 0.081±0.068 |
| LucaOne (G36.8M) | 0.062 | 0.074±0.065 | **0.544** | 0.551±0.042 | 0.079 | 0.100±0.079 |
| LucaOne (D17.6M) | **0.066** | **0.088±0.082** | 0.543 | **0.559±0.055** | **0.091** | **0.118±0.105** |
| **Class II RNA LMs** |  |  |  |  |  |  |
| 3UTRBERT (K3) | 0.022 | 0.053±0.055 | 0.517 | 0.535±0.037 | 0.043 | 0.066±0.065 |
| 3UTRBERT (K5) | 0.055 | 0.063±0.047 | 0.533 | 0.542±0.035 | 0.065 | 0.082±0.066 |
| UTR_LM | 0.049 | 0.071±0.068 | 0.533 | 0.551±0.049 | 0.067 | 0.103±0.097 |
| 3UTRBERT (K6) | 0.056 | 0.064±0.050 | 0.537 | 0.541±0.034 | 0.073 | 0.082±0.065 |
| MP-RNA | 0.052 | 0.072±0.068 | 0.539 | 0.550±0.043 | 0.077 | 0.098±0.084 |
| SpliceBERT | 0.063 | 0.071±0.059 | 0.543 | 0.550±0.044 | 0.079 | 0.097±0.088 |
| PlantRNA-FM | 0.061 | **0.083±0.075** | 0.542 | **0.558±0.051** | 0.083 | **0.115±0.102** |
| 3UTRBERT (K4) | **0.075** | 0.076±0.059 | **0.548** | 0.550±0.037 | **0.093** | 0.100±0.077 |
| **Class I RNA LMs** |  |  |  |  |  |  |
| RNABERT | 0.047 | 0.065±0.063 | 0.534 | 0.544±0.041 | 0.067 | 0.090±0.086 |
| RNAErnie | 0.051 | 0.076±0.069 | 0.541 | 0.551±0.045 | 0.081 | 0.100±0.092 |
| ProtRNA | 0.055 | 0.059±0.042 | 0.536 | 0.539±0.026 | 0.084 | 0.082±0.052 |
| RiNALMo (micro) | 0.069 | 0.098±0.098 | 0.549 | 0.565±0.064 | 0.086 | 0.126±0.130 |
| Uni-RNA (L16) | 0.063 | 0.107±0.105 | 0.543 | 0.575±0.072 | 0.087 | 0.145±0.142 |
| DGRNA | 0.086 | 0.111±0.083 | 0.555 | 0.572±0.054 | 0.094 | 0.142±0.108 |
| LAMAR (4k) | 0.065 | 0.091±0.077 | 0.545 | 0.562±0.054 | 0.095 | 0.126±0.105 |
| RFamLlama (Large) | 0.090 | 0.090±0.060 | 0.551 | 0.559±0.042 | 0.097 | 0.118±0.090 |
| GenerRNA | 0.059 | 0.100±0.092 | 0.535 | 0.564±0.059 | 0.097 | 0.130±0.123 |
| RNA-FM | 0.075 | 0.095±0.085 | 0.550 | 0.569±0.057 | 0.103 | 0.140±0.116 |
| LAMAR (2k) | 0.062 | 0.079±0.065 | 0.552 | 0.557±0.046 | 0.104 | 0.108±0.082 |
| Uni-RNA (L12) | 0.077 | 0.111±0.100 | 0.556 | 0.577±0.065 | 0.106 | 0.151±0.139 |
| AIDO.RNA (650M) | 0.088 | 0.122±0.107 | 0.558 | 0.584±0.070 | 0.106 | 0.169±0.143 |
| RFamLlama (Base) | 0.078 | 0.087±0.064 | 0.556 | 0.558±0.046 | 0.110 | 0.123±0.096 |
| RiNALMo (giga) | 0.075 | 0.116±0.108 | 0.552 | 0.581±0.075 | 0.114 | 0.167±0.151 |
| Uni-RNA (L8) | 0.096 | 0.107±0.084 | 0.563 | 0.571±0.052 | 0.133 | 0.139±0.109 |
| RiNALMo (mega) | **0.101** | **0.125±0.108** | **0.571** | 0.584±0.073 | 0.135 | 0.168±0.149 |
| AIDO.RNA (1.6B) | 0.098 | 0.121±0.100 | 0.564 | **0.585±0.067** | **0.136** | **0.175±0.135** |

**Reference**

1. Devlin, J., Chang, M.-W., Lee, K. & Toutanova, K. BERT: Pre-training of deep bidirectional transformers for language understanding. in *Proceedings of the 2019 conference of the north American chapter of the association for computational linguistics: Human language technologies, volume 1 (long and short papers)* (eds Burstein, J., Doran, C. & Solorio, T.) 4171–4186 (Association for Computational Linguistics, Minneapolis, Minnesota, 2019). doi:10.18653/v1/N19-1423.

2. Notin, P. *et al.* ProteinGym: large-scale benchmarks for protein fitness prediction and design. in *Proceedings of the 37th international conference on neural information processing systems* (Curran Associates Inc., New Orleans, LA, USA, 2023).

3. Arora, R. *et al.* RNAGym: Benchmarks for RNA fitness and structure prediction. in *ICLR 2025 workshop on AI for nucleic acids* (2025).

4. Hopf, T. A. *et al.* Mutation effects predicted from sequence co-variation. *Nat Biotechnol* **35**, 128–135 (2017).

5. Salazar, J., Liang, D., Nguyen, T. Q. & Kirchhoff, K. Masked Language Model Scoring. in *Proceedings of the 58th Annual Meeting of the Association for Computational Linguistics* 2699–2712 (2020). doi:10.18653/v1/2020.acl-main.240.
